# Immunomodulatory and antitumor effects of type I interferons and their application in cancer therapy

**DOI:** 10.18632/oncotarget.19531

**Published:** 2017-07-25

**Authors:** Ruan F.V. Medrano, Aline Hunger, Samir Andrade Mendonça, José Alexandre M. Barbuto, Bryan E. Strauss

**Affiliations:** ^1^ Viral Vector Laboratory, Center for Translational Investigation in Oncology, Cancer Institute of São Paulo/LIM 24, University of São Paulo School of Medicine, São Paulo, Brazil; ^2^ Department of Immunology, Institute of Biomedical Sciences, University of São Paulo, São Paulo, Brazil; ^3^ Cell and Molecular Therapy Center, NUCEL-NETCEM, University of São Paulo, São Paulo, Brazil

**Keywords:** IFNAR1/2, JAK-STAT, apoptosis, necroptosis, immunogenic cell death

## Abstract

During the last decades, the pleiotropic antitumor functions exerted by type I interferons (IFNs) have become universally acknowledged, especially their role in mediating interactions between the tumor and the immune system. Indeed, type I IFNs are now appreciated as a critical component of dendritic cell (DC) driven T cell responses to cancer. Here we focus on IFN-α and IFN-β, and their antitumor effects, impact on immune responses and their use as therapeutic agents. IFN-α/β share many properties, including activation of the JAK-STAT signaling pathway and induction of a variety of cellular phenotypes. For example, type I IFNs drive not only the high maturation status of DCs, but also have a direct impact in cytotoxic T lymphocytes, NK cell activation, induction of tumor cell death and inhibition of angiogenesis. A variety of stimuli, including some standard cancer treatments, promote the expression of endogenous IFN-α/β, which then participates as a fundamental component of immunogenic cell death. Systemic treatment with recombinant protein has been used for the treatment of melanoma. The induction of endogenous IFN-α/β has been tested, including stimulation through pattern recognition receptors. Gene therapies involving IFN-α/β have also been described. Thus, harnessing type I IFNs as an effective tool for cancer therapy continues to be studied.

## INTRODUCTION

Type I interferons (IFNs) are pleiotropic immunomodulatory cytokines that were originally described based on their ability to interfere in the viral infection cycle [1], that is to say, activate protective antiviral machinery in infected cells, their neighbors and, on a systemic scale, in antigen presenting cells (APCs) and T lymphocytes [2]. The IFN family is sub-divided into three types of cytokines — type I, type II and type III — which differ in their protein sequence, function, producer cell and cognate receptor. In humans, type I IFNs contain 18 distinct members (13 subtypes of IFN-α and one for each IFN-β, IFN-ε, IFN-κ, IFN-τ and IFN-ω) that, interestingly, all bind to the same cognate receptor, composed of the IFN-α/β receptor 1 (IFNAR1) and IFNAR2 subunits [3, 4]. Unlike the other type I IFNs, IFN-α and IFN-β have much more established and known roles in immunity and our review will focus on them. There is only one type II IFN — identified as IFN-γ — which binds to the IFN-γ receptor 1 (IFNGR1) and IFNGR2 subunits and is mainly produced by CD4^+^ helper T lymphocytes and natural killer (NK) cells. Type III IFNs consist of IFN-λ1, IFN-λ2, IFN-λ3 and IFN-λ4, which bind to the IFN-λ heterodimeric receptor 1 (IFNLR1) and Interleukin-10 (IL-10) receptor subunit β [3, 4].

During the past decades, a growing body of evidence clearly indicates that type I IFNs also play a pivotal role in naturally occurring and therapy induced immune responses to cancer [5]. This conclusion is based on two key observations: First, *Ifnar1* knockout (KO) mice are more tumor-prone upon exposure to the carcinogen methylcholanthrene (MCA) in comparison with mice that have functional type I IFN signaling. Second, tumors that arise from this IFN-α/β deficient context were more immunogenic (i.e., immune rejected when transplanted into a immunocompetent, naïve, syngeneic host) than when they were originated in the wild type background, thus demonstrating a significant role for type I IFNs in immune surveillance during carcinogenesis and tumor progression [6].

The ability of the immune system to eliminate nascent transformed cells, control and sculpt the immunogenicity of developing tumors while in a state of equilibrium, and upon escape of the immune control, exert pro-tumor functions, are all contemplated in the cancer immunoediting hypothesis [7, 8]. And among the cellular and molecular pathways identified so far, type I IFNs seem to be critical components for the host immune response against tumor, more specifically for the dendritic cell (DC) compartment [6, 9].

First identified by Steinman and Cohn [10, 11] DCs are professional APCs that act as central regulators of the antitumor immune cycle [12]. While in the steady state, DCs are present in their immature form, characterized by high capacity to capture antigens, but low secretion of cytokines and expression of co-stimulatory molecules (e.g., CD80, CD40, CD86). Yet, in the face of tissue injury, cell death or microbial infection, DCs are activated and migrate to the draining lymph nodes (LN) where they acquire fully mature phenotype (i.e, high expression of major histocompatibility complex (MHC) molecules and co-stimulatory signaling potential). DCs will then pass on the message received in the microenvironment where the antigen was encountered, delivering both antigenic (through MHC-I and MHC-II, due to their cross-presentation ability) and co-stimulatory signals, via membrane and secreted molecules, such as CD80, CD86 and IL-12, respectively, to prime naïve T cells [13, 14]. Interestingly, spontaneous immune responses to tumor cells have been shown to depend on the activation of DCs by type I IFNs [15] and as a result, one of the first cancer immunotherapies ever to be approved by the US Food and Drug Administration (FDA) consisted of high doses of recombinant IFN-α2b for melanoma and renal cell carcinoma [16]. Since then, numerous other antitumor strategies have exploited the immunomodulatory properties of type I IFNs to bring the full force of the immune system to the cancer fighting arena. For these reasons, in this review we will discuss the pleiotropic effects of type I IFNs on cancer and immunity and some of the therapeutic opportunities based on this critical interaction.

## DENDRITIC CELL SUBSETS IN CANCER

All DCs originate from bone marrow hematopoietic stem cells through sequential steps of differentiation that first form a common progenitor of macrophages/DCs and, secondly, give rise to two lineage specific precursors, one for monocytes and the other for DCs. The latter finally branches out into two major subsets, plasmacytoid DCs (pDCs) and conventional DCs (cDCs), which are further divided into cDC1 and cDC2, based on the transcription factors that drive the development process, cell surface markers and functions [17]. It is important to stress that much of the following nomenclature was obtained from studies of the mouse immune system and not all data from murine models perfectly match with their human counterpart.

In the mouse, cDC1s are negative for the CD11b marker, dependent on the inhibitor of DNA binding 2 (ID2), interferon regulatory factor 8 (IRF8) or basic leucine zipper ATF-like transcription factor 3 (BATF3) transcriptional factors, express the X-C Motif chemokine receptor 1 (XCR1) and display a remarkable capacity to cross-present antigens on MHC-I to activate CD8^+^ T cell responses. Among the BAFT3 driven DCs, CD8α^+^ DCs are localized in lymphoid organs, such as spleen and LN, thus not found in the non-lymphoid organs, whereas CD103^+^ cDCs are found in non-lymphoid organs [18, 19]. Importantly, *Baft3* KO mice, which lack both CD103^+^ and CD8α^+^ cDCs, when transplanted with highly immunogenic tumors (i.e., spontaneously regresses after being inoculated in immunocompetent mice) are not able to reject them [20] and even more critically, do not respond to checkpoint blockade immunotherapy [21]. On the other hand, cDC2s that can induce innate lymphoid cells (ILCs) and a Th2 immune response against multicellular parasites are a CD11b^+^ heterogeneous population, dependent on the IRF4 and zinc finger E-box binding homeobox 2 (ZEB2) transcription factors, express the signal regulatory protein alpha (SIRPα/CD172a) transmembrane protein and present MHC-II antigens to CD4^+^ T cells [15, 18]. Yet, their role in cancer remains unclear [15, 18, 19].

In humans, equivalent BAFT3 dependent DCs are identified by the expression of CD11c, CLEC9A, XCR1 and CD141 [22], have been found in different tumor types and, just as the murine DC, seem to be relevant in anti-tumor responses, since their presence correlates with a superior outcome in melanoma patients [14, 23]. Key insights on why these CD103^+^/CD141^+^ DCs display such unique function came from a recent work that used a mouse model of cancer to question which of the different tumor associated APCs (resident CD11b^+^, migratory CD11b^+^, CD8α^+^, CD103^+^ and macrophages) could phagocytose ovalbumin (OVA) and m-cherry from tumor cells, migrate to the draining LN and still be positive for m-cherry fluorescence, indicating the presence of the intact antigen. Remarkably, only in the CD103^+^ DCs subset could m-cherry fluorescence be detected. Upon isolation of APCs from the draining LN, once again, just CD103^+^ DCs were able to drive T cell responses against the OVA antigen. Furthermore, it was shown that C-C motif chemokine receptor 7 (CCR7) is required for CD 103^+^ DCs to traffic tumor antigens to the LN and that CCR7 levels correlate with T cell infiltrate and patient survival [24].

The other important subset is comprised of pDCs, whose development is driven by the E2-2 transcription factor and, curiously, morphologically resemble plasma cells. The pDCs specialize in producing and secreting large amounts of IFN-α after pathogen stimulation of toll-like receptor 7 (TLR7, detects single stranded viral RNA) and TLR9 (double stranded DNA), thus having a relevant role in the innate immune response against viruses [15, 18, 19]. In mice, pDCs are mostly found in blood and spleen, categorized by the expression of B220, Ly6C and the plasmacytoid dendritic cell antigen-1 (PDCA-1) markers and in humans, while negative for T, B and NK cell markers, pDCs are positive for CD4, CD123 (IL-3R), CD303 (BDCA-2), immunoglobulin-like transcript 3 (ILT3), and ILT7] [25]. Though the available data do not show a clear role for them in antigen presentation and initiation of the adaptive immune response, they are able to present antigens in the context of MHC-II molecules [15, 18, 19]. Indeed, upon activation by viruses, cytokines such as IL-3, CD40L or CpG oligonucleotide pDCs differentiate into full DC morphology and activate CD8^+^ and CD4^+^ T cell responses [26, 27]. Interestingly, in different types of tumors, including melanoma and prostate cancer, pDCs have been shown to be present in reduced frequency in the circulation, as they express multiple chemokine receptors, such as CXCR4 and ChemR23, that determine their tropism for sites undergoing pathological processes [28, 29], a subject thoroughly reviewed in [27] and [28].

However, in spite of suggestions that they may be involved in the initiation of the response by producing IFN-α in the tumor microenvironment, their role is still not convincing and, actually, some human studies have even associated pDC infiltration of tumors with poor survival [14, 30]. Accordingly, for reasons not fully understood, tumor-associated pDCs display a reduced responsiveness to TLR9 stimulation, become defective in IFN-α production and secrete immunosuppressive factors (e.g., IL-10) that along with regulatory T cells participate in immune surveillance escape, hence, favor tumor progression [27, 31]. Along the same lines, Le Mercier and colleagues used an orthotopic murine mammary tumor model to show that depletion of pDCs retarded tumor growth, evidencing their pro-tumor role. Remarkably, instead of TLR9, intratumoral administration of TLR7 ligand activated tumor associated pDCs and provoked a strong tumor regression effect [32]. Depletion of pDC and neutralization of type I IFNs prevented this outcome. Nevertheless, production of IFN-α by human pDCs can also be negatively regulated through the receptors BDCA2, NKp44 and ILT7, although only ILT7 has a known ligand, BST2 found on the cell membrane upon exposure to type I IFNs and also in a fraction of melanomas [27, 33].

Monocyte derived DCs (moDCs), or inflammatory DCs, originate from circulating monocytes that are thought to be drawn to the inflammatory cancer microenvironment since they are not present in a steady state [34]. moDCs express the Ly6C marker in mice or CD14^high^ in humans, but since in the mouse they are also positive for MHC-II, CD11b, CD11c and F4/80, it is hard to discriminate them from other CD11b^+^ populations or macrophages. Currently, CD64 (high-affinity IgG receptor gamma chain FcγRI) and MAR-1 (high-affinity IgE receptor FcεRIα chain) staining is used to discriminate moDCs from CD11b^+^ cDCs [14, 15, 34]. The capacity of moDCs to activate näive T cells requires further elucidation, since depending of the context and cytokines that are present, Ly6C^+^ monocytes can give rise to both macrophages and DCs [15]. Additionally, in tumors, Ly6C^high^ monocytes can remain as a heterogeneous population called myeloid-derived suppressor cells (MDSC) and through up-regulation of nitric-oxide, arginase, prostaglandin-E2 and production of transforming growth factor β2 (TGF-β2) can deeply impair function of effector immune cells [14, 35].

Regarding the infiltration of other DC subtypes into the tumor mass, data indicates that cDCs represent a scarce population, and as such are likely competing with other more abundant myeloid populations, such as macrophages and monocytes, for antigen capture and priming of T cells [36, 37]. In mouse models of cancer, the localization of most DC subtypes have been shown to be mostly localized in the tumor margins, with limited infiltration into the center [38]. In humans, owning to the difficulty to characterize the DCs *in situ*, their scarcity as well as cancer related heterogeneity, the localization of DCs remains poorly studied. Even so, in melanoma, peritumoral DCs have been observed which a more mature phenotype than infiltrating DCs’ [39, 40]. However, in a recent breakthrough, Lavin and collaborators aiming to determine the immune landscape of early lung adenocarcinoma lesions, used a multiscale immune profiling strategy based on mass cytometry by time of-flight (CyTOF) combined with single-cell transcriptomics and multiplex tissue imaging and observed that CD141^+^ DC (categorized by the high levels of CD207, CLEC9A, and XCR1) are significantly depleted in comparison with non-lung adenocarcinoma tissue, whereas CD1c^+^ DCs (expressing CD1c, CX3CR1 and IRF4) were observed more frequently [41]. However, the impact of tumor infiltrating DCs on clinical outcome needs further investigation, as other cells present in the tumor stroma are also playing a role, a topic that was thoroughly discussed in [40].

## TYPE I INTERFERONS SIGNALING PATHWAYS

In humans, the type I IFN family includes proteins encoded by at least 13 IFN-α genes (IFN-α1, -α2, -α4, -α5, -α6, -α7, -α8, -α10, -α13, -α14, -α16, -α17 and -α21) and one gene each for IFN-β, IFN-ε, IFN-κ, IFN-ω and IFN-τ [42]. Interestingly, IFN-α genes share 70–80% sequence homology and have about 35% identity with the IFN-β gene [43]. All type I IFN genes lack introns and are located on the short arm of chromosome 9 in humans and chromosome 4 in mice. IFNs-α and IFN-β have 186–190 amino acids, but they have a peptide that signals cleavage resulting in secreted proteins of 165 or 166 amino acids with the amino terminal domain being important for biological activity [44].

The various type I IFNs have differential tissue expression and although they bind to the same receptor (IFNAR1/IFNAR2) and signal through similar mechanisms [4, 45], they have different binding affinities and, consequently, give rise to different antiviral, antiproliferative, and immunomodulatory outcomes [46–48]. IFN-β has a ∼50-fold higher receptor-binding affinity to IFNAR1 than IFN-α [49], resulting in a more potent antiproliferative and perhaps distinct immunoregulatory action [47]. Interestingly, only IFN-β, but not IFN-α, stimulation enables the co-immunoprecipitation of IFNAR1 and IFNAR2 subunits [50]. Also, the IFNAR2 subunit binds type I IFNs with relatively higher affinity than IFNAR1, but the latter is absolutely required for signal transduction [42, 51, 52].

**Figure 1:**
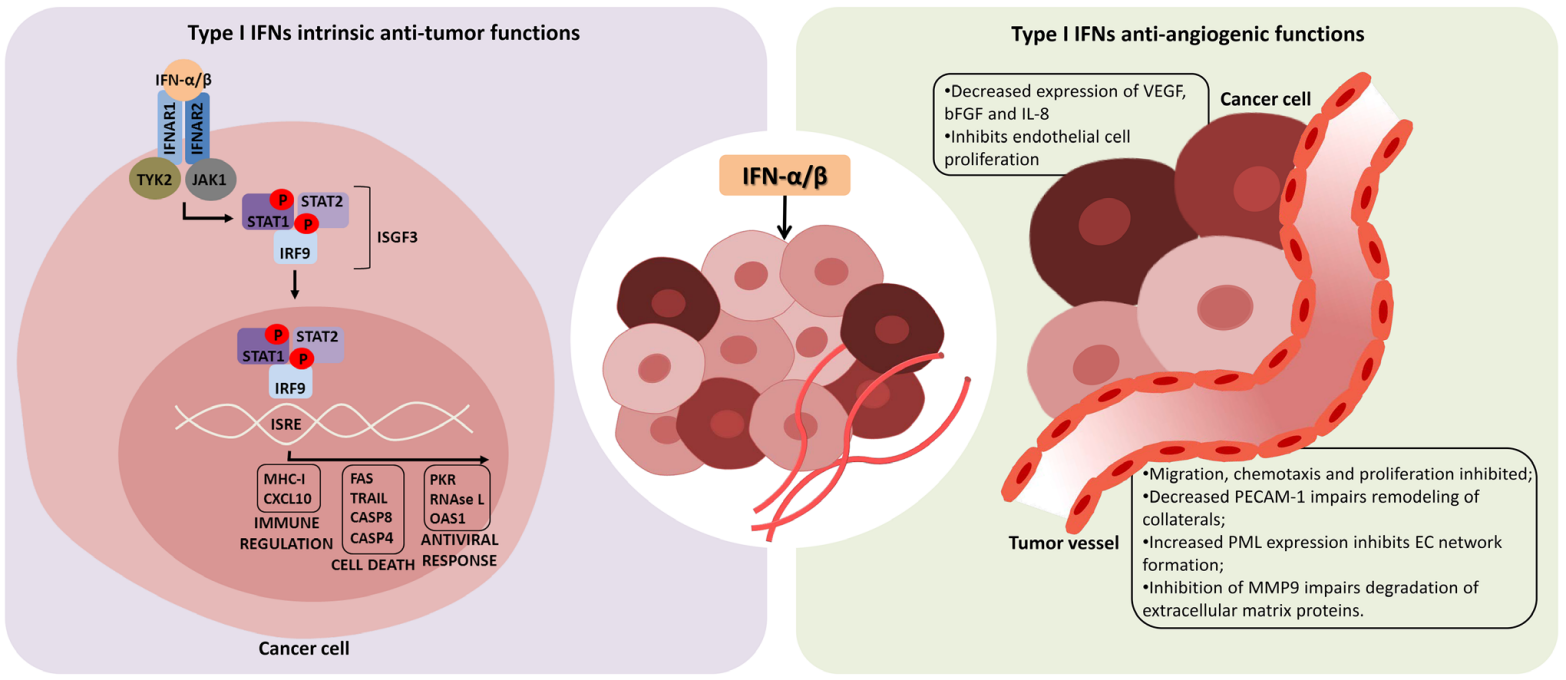
Intrinsic anti-tumor and anti-angiogenic functions of type I interferons Interferon-α/β (IFN-α/β) has direct effects in tumor cells inducing growth arrest and cell death (left panel). After binding to the heterodimeric IFN-α/β receptor 1 and 2 (IFNAR1/IFNAR2), type I IFNs induce a cascade of intracellular events which culminates in expression of genes whose promoters contain the IFN-stimulated response element (ISRE). In this way, several immuno-regulatory cytokines, cell death factors and proteins related to antiviral response are produced, as well as more IFN-α/β, which in turn affects neighboring cells. In addition, anti-tumor effects of type I IFN may also be a consequence of its anti-angiogenic function, impairing tumor vessel formation and leading to death of tumors by lack of oxygen and nutrients (right panel). IFN-α/β can inhibit the production of angiogenic factors by tumor cells, and also directly affects endothelial cells (EC), inhibiting their proliferation and secretion of factors responsible for EC chemotaxis and remodeling of extracellular matrix. Tyrosine kinase 2 (TYK2), Janus kinase 1 (JAK1), signal transducer and activator of transcription (STAT), IFN-regulatory factor 9 (IRF9), IFN-stimulated gene factor 3 (ISGF3), vascular endothelial growth factor (VEGF), basic fibroblast growth factor (bFGF), interleukin-8 (IL-8), platelet endothelial cell adhesion molecule-1 (PECAM-1), promyelocytic leukemia protein (PML), matrix metalloproteinase 9 (MMP9).

The IFNAR1/IFNAR2 receptor consists of transmembrane proteins which lack intrinsic kinase domains. They associate with a family of nonreceptor cytoplasmic tyrosine kinases, the Janus kinases (JAK1 and TYK2), so they can phosphorylate specific tyrosine residues of signal transducer and activator of transcription (STAT) proteins [53]. TYK2 associates with IFNAR1 while JAK1 acts in association with IFNAR2 [52, 54] on the inner side of the membrane, thus providing stability to the receptors and facilitating their cell surface localization, while serving as key components of signaling complexes [55, 56]. These complexes phosphorylate Tyr701 in STAT1α and in its spliced variant STAT1β, and Tyr690 in STAT2, enabling p-STAT to form heterodimers via their Src homology 2 (SH2) domains (Figure 1) [57, 58].

Once phosphorylated, the heterodimer STAT1/STAT2 binds to IRF9, forming the IFN-stimulated gene factor 3 (ISGF3) transcription factor complex [59]. The ISGF3 complex translocates to the nucleus and binds to *cis*-acting IFN-stimulated response elements (ISREs) in the promoters of IFN-stimulated genes (ISGs). While STAT1 and STAT2 require phosphorylation to be active, IRF9 functions as a DNA adapter molecule independently of its posttranslational modification induced by IFNα/β [60, 61].

Expression of type I IFNs is intimately connected and influenced by IRFs, a family of nine transcription factors with a similar DNA binding domain in their N-termini. IRF1 is expressed constitutively and also in response to IFN-γ, like IRF8, and it may determine which species of IFNs are induced by TLR activation. IFN-β induces synthesis of IRF7, which amplifies the synthesis of IFNs together with the constitutive expressed IRF3 [62, 63], and induces transcription of IFN-α genes [64].

In the type II IFN-γ signaling pathway, the homodimer of tyrosine-phosphorylated STAT1 binds to Gamma-Activated Sites (GAS) in ISG promoters [65]. Type I IFNs can also induce this pathway, triggering expression of genes with GAS in their promoters. Also, STAT1 can form heterodimers with other STATs leading to activation of other pathways (Figure 1) [66].

### Differences between IFN-α and IFN-β and their antitumor effects

Type I IFNs can induce expression of different genes depending on their concentration. Some genes are highly sensitive and require low picomolar concentrations, while other genes require 100-fold higher IFN-α/β concentrations for activation. Microarray analysis of expressed genes revealed that antiviral activity genes (such as Mx dynamin like GTPase 1 - *Mx1*, protein kinase R - *PKR* and 2′-5′-oligoadenylate synthetase 2 - *OAS2*) are induced by low amounts of IFN-α/β, whereas genes related to cell proliferation, chemokine activity and inflammation (like *IL-6*, C-X-C motif chemokine ligand 11 - *CXCL11*, and tumor necrosis factor related apoptosis inducing ligand - *TRAIL*) need a stronger IFN-α/β signal in order to be activated [47, 48, 67, 68]. The difference between antiviral and antiproliferative activities for IFN-α2 was 1,000-fold, while for IFN-β it was only 50-fold in WISH cells [51].

IFN-β binds to IFNAR1/IFNAR2 with higher affinity and thus forms more stable ternary complexes than IFN-α [47, 48, 69]. Because of this, IFN-β regulates cellular functions at concentrations that are orders of magnitude lower than any IFN-α subtype. Yet, all type I IFNs can induce antiviral responses at picomolar concentrations [69, 70].

In a study performed by Jaitin and collaborators, the authors engineered an IFN-α2 triple mutant (with H57A, E58A and Q61A mutations) that binds IFNAR1 with a 30-fold higher affinity than the wild-type protein and thus comparable to the binding affinity of IFN-β to IFNAR1. They observed that the HEQ mutant exhibited several functional characteristics of IFN-β, like similar patterns of gene induction and therefore substantially increased antiproliferative activity, without altering antiviral activity and ISGF3 formation. In this way, they indicated that functional differences between IFN-α2 and IFN-β are mainly due to their different binding affinities for IFNAR1. Therefore, the differential phenotypes of IFN-α2 and IFN-β are not qualitative, an observation that applies to both biological activities and gene induction patterns [47].

Also, the concentration of IFNAR1/IFNAR2 in the plasma membrane is critical to determine the cell sensitivity to type I IFNs. While IFN-β is more potent than IFN-α2 to induce antiproliferative activity in cells with native receptor numbers, IFN-α2 was equally able in cells with highly increased receptor numbers [46]. This may explain why IFN-β, but not IFN-α2, provides long-term signaling [70]. In Daudi cells, picomolar concentrations of type I IFNs are already enough to cause growth arrest because these cells have higher levels of the interferon receptors [51].

A recent report has suggested that tumor cells have lower interferon receptor levels, making them resistant to the antiproliferative activity of type I IFNs [71]. At low IFNAR1 concentrations, the complex is formed only with IFN-β, which binds IFNAR1 tightly, but not with IFN-α2 [72]. The duration of IFN stimulus is also critical to the decision between the antiviral state or antiproliferative response. While induction of antiviral activity requires a few hours of IFN-α or IFN-β exposure, antiproliferative activity requires days of constant IFN-α/β binding [70].

### Alterations in the type I interferon pathway in cancer

Since type I IFNs play a central role in the tumor microenvironment, especially with regard to anti-tumor activities, genetic alterations in this pathway are expected to be detrimental to prognosis and responses to therapy. The interferon gene cluster, found on human chromosome 9p21, encodes nearly all of the IFN genes and pseudogenes [43]. Interestingly, the cyclin dependent kinase inhibitor 2A (*CDKN2A*) gene also resides in this same region, encoding the p14^ARF^ (alternate reading frame, a functional partner of p53) and p16^INK4a^ (inhibitor of CDK4/6, thus an activator of retinoblastoma - Rb) proteins [73]. As such, 9p21 deletions could impact the p53, Rb and interferon pathways together or individually, depending on the exact nature of the deletion.

Homozygous deletion of IFN-α/β has been reported in leukemias, such as chronic myeloid leukemia (CML) and acute lymphoblastic leukemia (ALL) [74–78], and may be associated with resistance to IFN-α [79]. However, other studies indicate that a 400kb deletion including p16^INK4a^, but not the IFN gene cluster, is critical in lymphoblastic leukemias [80]. Deletion in the gene encoding IFN-α has also been correlated with post-transplant lymphoproliferative disorder [81]. Homozygous deletion of IFN-α contributes to the recurrence of head and neck squamous cell carcinoma (HNSC) [82]. The loss of the gene encoding IFN-β has been observed in glioma [83–85]. While for melanoma, hemizygotic deletion of 9p21 has been reported, observed in 12 of 14 cases [86], but this may reflect loss of *CDKN2A* alone or in combination with IFN-β [85]. Studies in a variety of cancer cell lines also support the notion that loss of the genes encoding IFN-α/β is a frequent event, though co-deletion of p16^INK4a^ and/or p14^ARF^ must be specifically examined [87–89].

Alterations in the interferon gene cluster are certainly not the only mechanism by which the type I IFN pathway may be disrupted. At least in cell lines, loss of STAT2 is associated with reduced apoptosis in response to IFN-α treatment [90]. Interestingly, methylthioadenosine phosphorylase (*MTAP*) also resides on chromosome 9p21 and can be epigenetically silenced in melanoma, resulting in impaired STAT1 signaling and serving as a marker of response to IFN-α therapy [91]. Infection with human papillomavirus (HPV), whether of high or low risk subtypes, has been correlated with resistance to IFN-α [92, 93]. Stimulator of interferon genes (STING) acts in the endoplasmic reticulum and promotes the transcriptional functions of nuclear factor-ĸB (NF-ĸB) and IRF3, thus playing a major role in anti-viral response [94]. In established melanoma and colorectal cancer cell lines, STING signaling is repressed due, typically, to epigenetic silencing of cyclic GMP-AMP synthase (cGAS) or STING itself. Loss of STING and/or cGAS was confirmed in some 54% of human colorectal cancer (Stage II-IV) while loss of both was seen in 41.7% of advanced stage human melanoma samples [95, 96].

Type I IFN and its receptor (IFNAR1/2) also contribute to the immunosuppressive tumor microenvironment. The paradox between inflammation and immunosuppression, especially with regard to type I IFN, was reviewed recently [97]. Expression of type I IFN is expected to promote the immune response, but it can also lead to the expression of indoleamine 2,3-dioxygenase (IDO), programmed cell death-ligand 1 (PD-L1) and IL-10, culminating in immunosuppression, as discussed here and in the review by Snell et al. [97]. In a recent report, Katlinski et al. [98] showed that IFNAR1 expression is reduced in colorectal cancer microenvironment, specifically in cytotoxic T lymphocytes, contributing to an immune-privileged niche that supports tumor growth. Restoration of IFNAR1 expression in the T cells was associated with renewed control over tumor growth [98]. The magnitude and duration of type I IFN signaling may be critical points to consider when designing therapies aimed at this complex pathway since sustained inflammation may lead to immunosuppression.

## ANTITUMOR FUNCTIONS OF TYPE I INTERFERONS

### Inhibition of cell growth and induction of apoptosis

The effects of type I IFNs on tumor cells may vary depending on the type of tumor and even more so when considering the specific cell in question. For example, IFN-β has a stronger antitumor effect than IFN-α in the early stage hepatocelular carcinoma (HCC) in patients with chronic hepatitis C. While IFN-α has been shown to induce apoptosis in HCC cell lines [99–101], Murata and co-authors showed that IFN-β had a superior antiproliferative effect as compared to IFN-α on three HCC cell lines, inducing cell cycle change and apoptosis, and more strongly upregulating ISGs, like Fas antigen and human leukocyte antigen (HLA)-class I molecules [102].

A time course study in WM9 melanoma cells with IFN-β (500 units/ml) identified more than 30% terminal deoxynucleotidyl transferase dUTP nick end labeling (TUNEL)-positive cells at 96 h, while IFN-α2 did not result in any positive staining. Other melanoma cell lines revealed similar sensitivity, like FemX cells, while Guilliams cells were partially sensitive and A375 cells were relatively resistant to either IFN-α2 or IFNβ, even at higher doses (up to 1000 units/ml) [103]. In another study with melanoma cells, IFN-β potency was also greater than IFN-α2 for induction of ISGs, like cytomegalovirus-induced gene 5 (*CIG5*/*viperin*), *CIG49*, *ISG54*, *TLR3*, *CXCL10*, *TRAIL*, as seen by microarray analysis of both WM9 and WM35 cell lines, while for the IFN-β-sensitive WM9 cell line, IFN-β also induced expression of *SP100*, tumor necrosis factor-stimulated gene 6 (*TSG6*), augmented in prostate carcinoma gene (*AIPC*), *Cyclin-E*, ubiquitin E2-like (*UBEL-2*) and ubiquitin-specific protease (*USP18*), as seen by RT-qPCR [104].

SK-MEL-2 and SK-MEL-24 cell lines were also more sensitive to the anti-proliferation effects of IFN-β than those of IFN-α2b *in vitro*. Matrigel invasion of SK-MEL-24 was significantly inhibited by both IFN-α2b and IFN-β and treatment of SK-MEL-24 with IFN-α2b or IFN-β decreased vascular endothelial growth factor-C (VEGF-C) and VEGF receptor-3 (VEGFR-3) protein expression. In a human melanoma xenograft model, SK-MEL-24 cells were injected intradermally in mice and tumor growth was reduced after IFN-α2b or IFN-β treatment. LN metastases were more frequent in mice treated with IFN-β than with IFN-α2b. One of six mice showed LN metastasis in the IFN-α2b group compared to three of six mice in the IFN-β group. Tumors were evaluated and revealed that both IFN-α2b and IFN-β decreased cell proliferation and increased the number of apoptotic cells, yet these effects were superior in IFN-β treated tumors. Also, VEGF-C/VEGFR-3 levels were reduced in tumors treated with IFN-α2b or IFN-β, but LYVE-1 was decreased only in IFN-α2b treated tumors, representing less intratumoral and peritumoral lymphatic vessels [105].

Cell lines that were relatively resistant to inhibition of cell growth by IFNs, including U937 (histiocytic lymphoma), HeLa (HPV-infected cervical adenocarcinoma), and T47D (ductal breast carcinoma) were also not TUNEL-positive in response to IFN-α2 or IFN-β. Other cell lines, like ACHN (renal cell carcinoma), Minors (melanoma), NIHOVCAR3 (ovarian carcinoma) and MCF-7 (breast carcinoma) had an increase in TUNEL-positive cells in response to IFN-β but not IFN-α2 [103].

Chen and colleagues showed that both IFN-α and IFN-β induced apoptosis in U266, RPMI-8266, and NCI-H929 multiple myeloma cell lines and plasma cells from 10 patients with multiple myeloma [106]. Expression of TRAIL, which contains 2 IFN-stimulated regulatory elements in its promoter [107], seems to be the main event for cell death induction, followed by caspase-8 activation, Bid cleavage, cyt c release and caspase-3 activation [106].

In a study performed by Rozera and co-authors, TS/A adenocarcinoma cells were injected with retrovirus encoding IFN-α or IFN-β and these cells were inoculated in BALB/c mice. More host-infiltrating cells were observed in TS/A-IFN-α and TS/A-IFN-β than in parental TS/A tumors (macrophages, granulocytes, and lymphocytes, being CD8^+^ T cells more numerous than CD4^+^ T cells). Also, fewer blood vessels were observed in TS/A-IFN-α or TS/A-IFN-β tumors as compared with parental TS/A tumors, being the vasculature of TS/A-IFN-β tumors scarcer than TS/A-IFN-α tumors, even when no differences in the expression of angiogenic factors (VEGF and basic fibroblast growth factor - bFGF) were found. However, the expression of pro-inflammatory cytokines, such as IL-1β, IFN-γ and tumor necrosis factor-α (TNF-α), was higher in TS/A-IFN-α than TS/A-IFN-β tumors and absent parental TS/A tumor. Finally, survival of TS/A-IFN-β mice that produced higher levels of IFN-β or TS/A-IFN-α mice was three- to four fold longer than the control group, while metastatic ability of TS/A cells was reduced in mice injected with either TS/A IFN-α or IFN-β cells [108].

### Inhibition of angiogenesis

Angiogenesis is an important antitumor therapeutic target because it is required for tumor growth [109, 110] for the delivery of oxygen and nutrients to the fast-growing tumor cells [111]. As shown in Figure 1, the anti-proliferative and cell death inducing functions of type I IFNs also inhibit angiogenesis [112, 113]. For example, after IFN-α/β treatment, tumor vessels undergo necrosis [113]. IFN-α/β also prevents tumor cell production of angiogenic growth factors, like bFGF [114–116], VEGF [117, 118], and IL-8 [119, 120]. Interestingly, when MBT-2 (murine transitional carcinoma of the bladder) or L1210R (murine leukemia resistant to the antiproliferative effects of IFN) cells were treated *in vitro* with IFN-β and then inoculated intracutaneously in C3H/He or Swiss mice, respectively, the inhibition of angiogenesis was noted within 24 hours of tumor cell inoculation, even before their antiproliferative effects on tumor cells [112]. Thus, inhibition of angiogenesis can be counted among type I IFN’s anti-tumor benefits.

McCarty and collaborators showed that endogenous type I IFN signaling is involved in the regulation of angiogenesis. These authors implanted sponges filled with proangiogenic molecules (bFGF, VEGF, and TGF-α) in mice deficient for IFN-α/β receptor and observed superior vascularization when compared to control mice with functional type I IFN signaling. Moreover, the antiangiogenic effects of type I IFNs resulted in inhibition of tumor growth in animal models [121].

It is known that in inflamed tissues, the release of IFN-α by leukocytes trigger macrophage activation, which then causes the release of TNFα. Both IFN-α and TNF-α induce accumulation of promyelocytic leukemia protein (PML) in HUVECs and in microvascular endothelial cells (HMVECs). PML was shown to be indispensable for TNF-α and IFN-α-mediated inhibition of EC network formation, but no significant differences in apoptosis of HUVEC treated with TNF-α, IFN-α or vehicle were detected [122]. PML is highly expressed in normal vascular endothelium and inflamed tissues [123] and known as an ISG [124] through STAT1 induction, since knockdown of STAT1 significantly impairs PML expression in these cells. The authors have shown PML suppressed integrin β1 (ITGB1) expression in both HUVECs and HMVECs, an important protein which regulates ischemic neovascularization [125], cell-to-cell and cell-to-extracellular matrix adhesion and cell migration [126].

Spaapen and others have observed that IFN-β–secreting B16 cells injected subcutaneously in mice had impaired growth, yet when implanted into mice lacking IFNAR1, tumors grew progressively. This shows that the antitumor effect of IFN-β was dependent on signaling via host cells and did not act directly on tumor cells, a situation that was also observed with IFN-α. This effect was also independent of adaptive immunity, since tumor regression was also observed in *Rag2*^*−/−*^γ*c*^*−/−*^ mice (deficient in T, B, and NK cells). However, a diminution of blood vessel density was observed in the IFN-β-secreting tumors. In the work, the authors showed that IFN-β has a direct effect on nonhematopoietic Tie2^+^ cells, that is to say, vascular ECs, causing inhibition of angiogenesis [127].

While IFN-β may reduce the number of tumor vessels, it can also contribute to vessel maturation. Dickson and others showed that treatment of human xenografts in immunodeficient mice with an adeno-associated virus (AAV)-vector encoding the human IFN-β gene resulted in maturation of the intra-tumor vasculature, yet inhibition of angiogenesis [128]. Also, treatment of tumors with IFN-β, encoded by an adenoviral vector, promotes an increase in inducible nitric oxide synthase (iNOS) and a decrease in bFGF and TGF-β1 levels [129], resulting in inhibition of tumor growth.

Indirectly, IFN-β inhibits matrix metalloproteinase 9 (MMP9) gene expression, which is responsible, together with MMP2, for degradation of extracellular matrix proteins collagen and elastin, a process required for initiating the enlargement of collateral vessels [130]. This is in accordance with Nelissen and others (2002), who have shown that IFN-β inhibited expression of MMP9 in monocytic and peripheral blood mononuclear cells [131] (Figure 1).

The mechanisms by which IFN-β inhibits tumor growth and angiogenesis may also involve tumor-infiltrating neutrophils. Jablonska and others showed that IFN-β-deficient mice presented faster tumor growth of injected tumor cells and larger tumors compared to wild-type mice. This was associated with an increase in tumor angiogenesis and tumor-infiltrating CD11b^+^Gr1^+^ neutrophils, which are responsible for expression of proangiogenic and homing factors. After treatment with IFN-β, these neutrophils had reduced gene expression of *VEGF*, *MMP9*, CX-C chemokine receptor type 4 (*CXCR4*) and the receptor for stromal-derived factor-1 (*SDF-1*), contributing to limiting tumor angiogenesis. Also, when neutrophils obtained from IFNAR-deficient mice were injected in wild-type mice, tumor growth was increased and accompanied by more mature vessels when compared with neutrophils obtained from WT mice [132].

Type I IFN may affect endothelial cell (EC) survival [133] and function, as indicated by inhibition of migration, chemotaxis and proliferation of HUVEC (human umbilical vein endothelial cells) [134], but as said before, although these cytokines share the same receptors, they exert different effects on tumor viability and angiogenesis. While there are several studies demonstrating the antiangiogenic effects of type I IFNs, there are few studies comparing the different effects of IFN-α and IFN-β, and even less including other members of the type I IFN family. In a study comparing type I IFNs, IFN-α2b inhibited *in vitro* vessel formation of HUVEC by 20%, whereas inhibition due to IFN-β was around 80%. *In vivo*, IFN-α2 inhibited vessel growth by 30% in SK-MEL-1 tumors, whereas IFN-β inhibited vessel formation by 80%. While both IFN-α2b and IFN-β inhibited HUVEC proliferation, neither of them was able to induce apoptosis. Genes induced by IFN-β in HUVECs are *p56*, *CXCL11*, *ISG20*, melanoma differentiation-associated-5 (*MDA-5*), HECT and RLD domain containing E3 ubiquitin protein ligase family member 6 (*HERC6*), *CXCL10*, SAM and HD domain containing deoxynucleoside triphosphate triphosphohydrolase 1 (*SAMHD1*), *p60*, *Sp100B* and monocyte chemoattractant protein 2 (*MCP-2*) [135]. This effect was also observed by Erdmann and others who described that IFN-β inhibited cell cycle and proliferation of human micro and macrovascular ECs, but did not induce apoptosis [136]. Interestingly, VEGF is responsible for phosphorylation of IFNAR1, followed by ubiquitination induced by the protein kinase D2 (PKD2), which results in the degradation of IFNAR1 and promotion of angiogenesis [137].

Albini and co-authors have compared the effects of retroviral vector-packaging cell lines encoding IFN-α or IFN-β cDNAs (α1Am12 and βAm12) upon ECs. In this study, both conditioned media from α1Am12 or βAm12 decreased chemotaxis and invasion of ECs, however only βAm12 inhibited EC differentiation into capillary-like structures on Matrigel. Also, IFN-β’s superiority in inhibiting angiogenesis was confirmed in an *in vivo* model, in which sponges containing a very potent angiogenic cocktail co-injected along with α1Am12 cells in C57BL/6 mice produced a limited inhibitory effect on angiogenesis, while βAm12 cells markedly impaired vessel formation. This was also observed in nude mice, confirming the effect of IFN-β on ECs independent of a T-cell response [138].

As will be discussed below, the first antitumor efforts involving type I IFNs were developed using IFN-α, but IFN-β later gained importance in this field as a result of studies showing its increased antitumor and antiangiogenic effects, although further investigation is needed to support the notion that IFN-β provides superior antiangiogenic activity. Studies comparing the gene expression profiles of ECs treated with different type I IFNs could be very enlightening as to the different effects of these cytokines on angiogenesis. Also, *in vivo* and *in vitro* treatments of ECs with different type I IFNs and posterior analysis of angiogenesis could reveal type I IFN’s functional effects. Finally, knockout models specific for each type I IFN could be used to confirm their individual functions, thus providing evidence based on endogenous proteins.

### Immunomodulatory and regulatory effects of type I interferons

**Figure 2:**
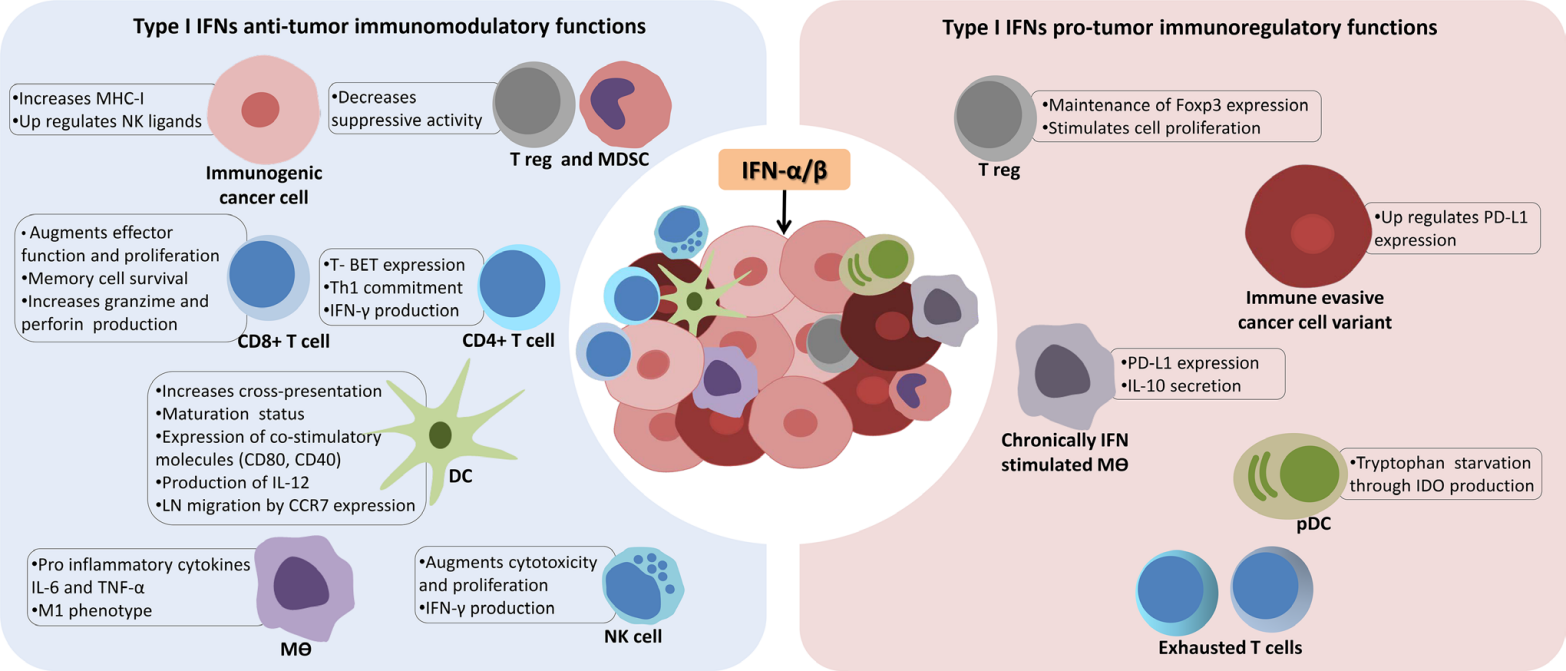
The context dependent and complex role of type I interferons in cancer immunity Activation or delivery of interferon-α/β (IFN-α/β) into the tumor microenvironment can result in immunomodulatory and regulatory functions. In the first scenario (left panel), in order to unleash an effective immune attack against cancer cells, type I IFNs modulate innate and adaptive compartments through multiple mechanisms to provide a pro-inflammatory context suitable for antigen recognition by tumor associated dendritic cells (DCs) and priming of T lymphocytes. Importantly, as type I IFNs enhance co-stimulatory molecules of DCs, they also increase the unique ability of DCs to cross-present phagocytized tumor antigens to CD8^+^ T cells. Additionally, immunogenic tumor clones (represented in pink) have their antigenicity increased by up regulating major histocompatibility complex class I (MHC-I) molecules. However, type I IFNs can also favor tumor progression and escape from immune control (right panel), especially under chronic exposure conditions, since they can induce macrophages (mϴ) to produce Interleukin-10 (IL-10), that along with tryptophan starvation mediated by indoleamine 2,3-dioxygenase (IDO) and expression of programmed death-ligand 1 (PD-L1) by immune evasive tumor cells (dark red), greatly impairs T cells functions. Tumor necrosis factor–α (TNF-α), plamacytoid DCs (pDCs), myeloid-derived suppressor cells (MDSC), regulatory T cell (T reg), C-C chemokine receptor type 7 (CCR7), natural killer cells (NK).

So far, we have exposed several anti-neoplastic functions attributed to type I IFN, but its main function is most often associated with immune modulation. Depending on the stimulus, both IFN-α/β can be produced by almost any cell type, including fibroblasts and leucocytes [139]. Such inducers act through pattern recognition receptors (PRRs) that sense pathogen-derived and non-pathogenic components, for example: double-stranded RNA (dsRNA) from RNA viruses detected by TLR3, both cytosolic DNA and second messenger cyclic di-GMP24 from bacteria by STING [5, 139], as well as danger-associated molecular patterns (DAMPs) released upon cellular stress or therapy induced cell death. Heil and Land suggest dividing mammalian DAMPs into five classes according to different PRRs. Class I DAMPs, like HMGB1 or heat shock proteins, are sensed by TLRs and trigger the MAPK signaling cascade. Class II DAMPs are perceived indirectly by the NOD-like receptor family protein 3 (NLRP3) inflammasome and comprise ROS, monosodium ureate, eATP and dsDNA. Both classes I and II are involved in maturation of DCs. Class III comprises stress-induced soluble major histocompatibility complex class I-related chains A/B (MIC-A/B) and UL-binding proteins (ULBPs) and are sensed by receptors such as NKG2D, expressed by innate lymphocytes, like NK cells, and innate-like T-lymphocytes, like gamma delta T-cells. Class IV represents neoantigens, such as non-muscle myosin-II (NMHC-II), actin cytoskeleton and oxidized phospholipids that, together with IgM antibodies, bind to classical lectin receptors and trigger activation of the complement cascade and alternative pathways. Finally, class V DAMPs are called Dyshomeostasis – Associated Molecular Patterns, which comprise altered pattern of molecules resulted from perturbations in the steady-state of the intra- and/or extracellular microenvironment, like hypoxia, changes in acidity or osmolarity, and metabolic stress [140]. Thus, type I IFNs may be released as part of the natural evolution of a disease or as a consequence of therapeutic interventions. However, in cancers, an important difference between these two scenarios is that the local immune suppressive environment modifies the expected “physiological” response, either counteracting or amplifying the immunomodulatory functions of type I IFNs (Figure 2).

Immunomodulatory effects triggered by type I IFNs can act on both innate and adaptive immune compartments [141]. In a temporal scale, macrophages can be considered as early producers of type I IFNs that act on nearby macrophages and other innate cells, such as NK, to provide a pro-inflammatory context (release of cytokines IL-6, TNF-α) suitable for antigen capture and presentation by tumor associated APCs and priming of immune effector cells [2, 142]. Macrophages, similarly to DCs, produce both type I and II IFNs, and upon activation display high levels of MHC class I and II in order to boost a T cell response, thus working as a link between innate and adaptive immunity [143]. Type I and II IFNs have also been show to polarize macrophages into an M1 immunostimulatory phenotype with anti-tumor functions, rather than an M2 phenotype, which may have pro-tumor activities [143, 144].

However, as part of an adaptive immune resistance mechanism that takes place after an inflammatory response, negative regulators of the immune response are induced, aiming to limit both duration and specificity of the immune attack [145]. Regulatory mechanisms induced after IFN-α/β production include secretion of IL-10 and expression of PD-L1 [146]. Both IL-10 and PD-L1 are well known inhibitors of CD8^+^ T function and, as shown by Shaabani and collaborators, upon infection with lymphocytic choriomeningitis virus (LCMV), type I IFNs are produced in high amounts by CD169^+^ macrophages to combat the virus, but as a consequence leads to up-regulation of PD-L1 and therefore CD8^+^ T cell exhaustion [147]. Lack of this CD169^+^ macrophage population, known for their unique distribution in secondary lymphoid organs and antigen handling capacity to prime CD8^+^ T cells, impairs viral control, IFN-α production, and eventually mice succumb, thus confirming the regulatory function type I IFNs on macrophages and CD8^+^ responses [147, 148]. Interestingly, in a more general view of the tumor microenvironment context, PD-L1 expressing tumor-infiltrating immune cells, including macrophages and DCs, in head and neck cancer patients have been shown to result in a more favorable prognosis than when PD-L1 is expressed on tumor cells [149]. Indeed, in a recent study by Noguchi and colleagues, IFN-γ was responsible for up regulating PD-L1 in tumor cells, contributing to immune escape, but unexpectedly, the majority of PD-L1 molecules were expressed by the host immune system, especially in the macrophage compartment, suggesting a mechanism in *trans* to impair T cells function. Intriguingly, after antibody mediated blockade of IFN-γ, levels of PD-L1 on tumor cells drastically decreased, but remained elevated on tumor associated macrophages, suggesting an additional mechanism not dependent on IFN-γ to induce PD-L1 [150]. The exact role for type I IFNs in mediating PD-L1 immune escape remains to be elucidated, but it is tempting to speculate that early production of IFN-α by APCs augments IFN-γ production by CD4^+^ T lymphocytes [151] and NK cells [152], and thus leads to PD-L1 up regulation in the tumor microenvironment.

Within the DC compartment, type I IFNs act as strong maturation signals, increasing expression of co-stimulatory proteins CD40, CD80, CD86 and MHC molecules [153], enhancing their unique properties to process and present apoptotic cell antigens through their scavenger receptor lectin-like oxidized-LDL receptor-1 (LOX-1) [154], and stimulating migration to the draining LNs by up regulating the CCR7 chemokine receptor [155]. Notably, together with the production of IL-12 and IL-23, all of these actions support Th1 and Th17 cytotoxic T lymphocyte (CTL) responses, including increased survival of CD8^+^ memory T cells and expression of granzyme B and perforin 1 [2, 5].

Among the different DC subsets, using bone marrow chimera experiments, Diamond and collaborators have demonstrated that *Ifnar1*^−/−^ CD8α^+^ DCs lose their capability to cross present tumor antigens and as a consequence, when regressor tumors are transplanted, the host is no longer able to immune reject them [9]. Cross-presentation is defined as the presentation of internalized antigens in the context of MHC-I molecules to CD8^+^ T cells, instead of the MHC-II context [5]. This data implies that this DC subset must be endowed with mechanisms to recognize stress signals from dying tumor cells, capture their antigens and present to CD8^+^ T cells, in order to trigger CTL responses. A plausible mechanism for the re-routing of capture antigens has been made by Reis and Sousa, investigating the C-type lectin domain family 9 member A (CLEC9A), a plasmatic receptor for necrotic cells that is highly expressed in CD8α DCs. They found that instead of activating these cells, CLEC9A was directing necrotic cell cargo into a recycling endosomal compartment, favoring cross-presentation to CD8^+^ T cells. Along the same lines, CLEC9A deficiency in CD8α^+^ DCs impaired their capability to prime CD8^+^ T cells but not CD4^+^ T cells, indicating that this plasmatic receptor was promoting cross-presentation of dead cell–associated antigens on MHC class I molecules. However the role of CLEC9A on the cancer immunity cycle needs further investigation [156]. Along the same lines, *Tmen173−/−* DCs, that lack the STING pattern recognition receptor gene, have also been found to be unable to prime CD8^+^ T cells [5, 157].

Taken together these data indicate that type I IFNs are involved in DCs antigen preseting functions, although immune regulatory mechanism are also triggered in order to limit the magnitude of the inflammatory response. Evidence of such regulatory mechanisms can be observed in pDCs, which have an unclear role in cancer, but are known to be a major source of type I IFNs. pDCs that infiltrate breast cancers have been shown to be defective in producing IFN-α and to co-localize with T regulatory cells (T-reg, CD3^+^CD4^+^CD25^+^ FOXP3^+^) [158], suggesting that either T-reg cells may be inhibiting IFN-α production or that pDCs somehow support the proliferation of T-regs. Indeed, mature pDCs can orchestrate tolerogenic immune responses through the induction of IL-10 IDO [159, 160], which catabolizes the essential amino acid tryptophan into a more stable metabolite, kynurenine and, as a consequence, stimulates T-reg proliferation while inducing CD8^+^ T-cell dysfunction, anergy and apoptosis [160, 161]. Catabolism of tryptophan in cancer is being recognized as a powerful suppressor of antitumor immunity since several tumor types were found to over-express IDO [162] and this was recently implicated as a critical mechanism of resistance to checkpoint blockade immunotherapy targeting the cytotoxic T lymphocyte antigen-4 (CTLA-4) pathway [163]. Interestingly, the IDO promoter contains transcriptional targets of both IFN-γ and IFN-α/β and in the case of pDC, both type I and type II IFNs were shown to be equipotent and exert additive effects on the induction of IDO [164].

In the lymphocyte compartment, type I IFNs have been show to act on both CD4^+^ and CD8^+^ T cells to dictate a Th1 immune response through the activation of STAT4 and T-bet expression, which is a T-box transcription factor expressed in CD4^+^ T lymphocytes committed to Th1 development [165, 166]. Interestingly, type I IFNs can also reverse the commitment of a Th2 humoral response by suppressing the GATA3 transcription factor, but when compared to IL-12, another Th1 inducing cytokine, it was shown that type I IFN cannot sustain T-bet expression by itself, needing other cytokines, such as IL-1β, to maintain this phenotype [166]. In terms of cancer, Th1 cells are known for orchestrating CTL responses that are implicated in the destruction of a tissue during autoimmune responses as well in antitumor responses [167], as demonstrated by a mechanistic degree of similarity shared between them [167, 168] and increased survival rate observed in patients with a CTL tumor infiltrate [169].

Another critical immunomodulatory mechanism induced by type I IFNs that directly affects T cell responses is the positive regulation of tumor antigens that are presented on cancer cells by MHC-I molecules, allowing the immune system to detect the tumor and distinguish it from a normal cell [170, 171]. In fact, up regulation of MHC-I by type I IFNs [172], has the potential to counteract the frequent down regulation of MHC-I found in human tumors resistant to immunotherapies [173]. Indeed, as recently demonstrated in a mouse model using tumors resistant to PD-L1 checkpoint blockade immunotherapy, high dose radiation directly upon the tumor mass induces a systemic increase of IFN-β levels and restores therapeutic efficacy by up-regulating MHC-I molecules in the tumor cells. Antibody mediated blockade of IFNAR1 completely abrogated this effect [174].

Furthermore, the regulation of immunity in cancer by IFN-α/β also involves increased cytotoxic functions of NK cells [146], which are lymphoid cells that through a balance of activating (e.g., NKG2D) and inhibitory receptors (e.g, killer-cell immunoglobulin-like, KIR) can effectively kill tumor cells [175]. Activation of the NK response was demonstrated to positively sustain an M1 macrophage phenotype and to edit tumor immunogenicity in a process independent of T cells [176]. And, curiously, type I IFNs inhibit the elimination of CD8^+^ T cells by NK cells [177], a phenomenon observed in conditions where NK cells assume a regulatory function over the adaptive immune response in order to prevent chronic inflammation and generation of auto-immune reactions [178].

Finally, in support of defining the appropriate context for an effective CTL response, type I IFNs were also shown to inhibit immune-suppressive actions of T-reg cells and MDSCs [144]. Yet in inflammatory conditions, it has been shown that type I IFNs were required for maintenance of Foxp3 expression and immune suppressive functions of T-regs, since transfer of T-regs obtained from *Ifnar1* KO mice were not able to inhibit the induction of the T-cell mediated colitis, as seen for T-regs obtained from wild-type mice. Indeed, administration of recombinant IFN-α reduced T cell-mediated colitis by increasing the number of T-regs and their suppressive functions [179].

As discussed here, the role of type I IFNs in immunity is complex and context dependent, assuming either antitumor or pro-tumor functions determined by the exposure to type I IFNs pre or post antigen encounter, if produced acutely or chronically, in low or high levels. For example, using an LCMV model, it was observed that early and transient production of IFN-α by pDCS exerts minimal effects on CD8^+^ T cell responses, but administration of recombinant IFN-α5 and IFN-β on days that coincide with endogenous type I IFNs decline, hence providing sustained stimulation, can prevent CD8^+^ T cell exhaustion and viral persistence [180]. Furthermore, this complex role of type I IFNs was evidenced in two complementary works both exploring a model of chronic LCMV infection, where IFN-α was transiently produced, yet ISG expression was prolonged. In the first work, it was shown that genetic or antibody mediated blockade of IFN-α signaling prior to infection leads to increased viral replication and loss of infection control, thus confirming the antiviral role of IFN-α [181]. Whereas, in the second, after establishment of the chronic infection, IFN-α blockade acted by reducing IL-10 and PD-L1 levels and, as a result, ameliorated T cell exhaustion and, even though it took 2 months, resulted in significantly lower virus titers [182]. Therefore, there seems to be a paradoxical function of type I IFNs: early (i.e., prior to antigen encounter) antiviral effects of type I IFNs are critical for host protection, promoting immune activation by stimulating an NK cell attack, enhancing DC antigen presenting function and favoring T cell proliferation, but after this adaptive immune response has been unleashed, chronic stimulation of the type I IFN pathway can result in immunoregulatory mechanisms that aim to shut down long lasting and unresolved immune responses, although as discussed below, therapeutically induced IFN-α/β can restart or reinvigorate a new immunity cycle. Examples of such duality are also observed during hepatitis C virus infection, in which strong IFN-α/β signature correlates with poor responses to therapy, as well as in chronic HIV infections and on latent *Mycobacterium tuberculosis* [183].

In cancer, the opposing role IFNs is better characterized with IFN-γ, especially on the induction of PD-L1 on cancer cells. As recently demonstrated by Benci and colleagues, prolonged exposure of tumors to IFN-γ induces a STAT-1 epigenetic signature as well as ligands for inhibitory receptors that results in PD-L1 dependent and independent mechanisms of resistance to checkpoint blockade immunotherapy [184]. Intriguingly, type I IFNs were also shown to be required for maintenance, not induction, of the PD-L1 independent resistance phenotype, but the precise contribution was not thoroughly explored [184]. Moreover, disrupting IFN-γ driven resistance with ruxolitinib, a JAK1/JAK2 inhibitor, renders CTLA-4 checkpoint blockade resistant tumors sensitive again. Accordingly, the work also provides clinical evidence that high expression of ISG and IFN-γ signaling is associated with tumor progression after PD-L1 therapy [184]. The mechanism that is behind the complex and opposing functions of IFNs is likely mediated by a qualitative and quantitative difference of regulators. For example, in a mouse melanoma model, therapeutic efficacy of high-doses of intratumoral IFN-α/β appears not to be T cell dependent, but rather relies on their anti-angiogenic properties, acting directly on the tumor vasculature [127]. Further studies that can dissect the molecular basis of this complex mechanism, specifically the influence of the producing cell, timing and magnitude, are surely needed.

## TYPE I INTERFERONS IN CANCER THERAPY

### IFN-α/β therapy for melanoma

Type I IFN therapy as treatment for melanoma utilizes the recombinant protein itself or as a complex with polyethylene glycol (PEG) in order to improve protein stability [185]. High dose IFN-α2b has been approved as an adjuvant therapy after surgical resection of cutaneous melanoma in patients with a high risk of death from recurrence. This approach is beneficial for improving disease-free survival, but the therapy itself is not well tolerated. Here we will address some of the progress and pitfalls of IFN-α/β therapy.

When caught early, surgical excision of melanoma can be curative. However, once disseminated, the treatment of melanoma is quite inefficient and survival rates are quite dismal [186]. The use of IFN-α2b was approved as an adjuvant therapy by the FDA in 1996 based on clinical findings that showed high-dose treatment was beneficial for prolonging relapse free survival and overall survival [187]. Unfortunately, the high-dose treatment is associated with severe adverse effects, including fatigue, myalgia, pyrexia and depression. While lower doses may decrease the adverse effects, they do not offer the same benefit in relapse free survival [188]. The use of PEG-IFN-α2b has been shown to reduce some of the fatigue and flu-like symptoms seen with the non-pegylated protein [189, 190]. In comparison, adjuvant therapy with IFN-β is standard practice in Japan where low-dose administration has been reported as beneficial for maintenance therapy [191].

High-dose IFN-α treatment has also been tested as a neoadjuvant for patients with locally advanced disease with the intention of reducing T-regs and improving CD8^+^ T cell memory [192]. Clinically, neoadjuvant IFN-α therapy was associated with increased intratumoral DC in 11/20 patients who showed objective clinical response [193]. For the treatment of disseminated melanoma, higher doses or continuous administration of IFN-β were met with limited efficacy and toxicity [194, 195]. Association of IFN-α with dacarbazine or other chemotherapies was not beneficial [192].

The results from several long term and large cohort trials exploring melanoma patient populations and treatment regimens have been published in the past few years. For example, in the Sunbelt Melanoma Trial, started in 1997, treatment of patients with sentinel lymph node involvement were treated with high dose IFN-α2b (HDI) with or without complete lymph node dissection and clinical progress was compared to patients who did not receive HDI. In this trial involving more than 900 patients, no clinical benefit was associated with the use of HDI [196]. Long term follow up of the EORTC 18952 trial was recently reported, revealing that a 13 month IFN-α2b treatment regimen was inferior to a 25 month regimen in patients with stage IIB-III melanoma, however the difference was marginal. Interestingly, ulceration of the primary tumor was associated with increased sensitivity to IFN-α2b [197]. Final analysis of the Dermatologic Cooperative Oncology Group Trial was reported in 2015, showing benefit of IFN-α2b treatment for relapse free survival, but not overall survival [198].

The study of melanoma treatment using recombinant type I IFN is ongoing and aims to identify patient populations that will benefit from this and other adjuvant approaches, including ipilimumab and vemurafenib [186, 197, 199–201]. Even so, the use of type I IFN for the treatment of cancer is certainly not limited to melanoma. For example, treatment of prostate carcinoma [202] and myeloproliferative disorders [203] with type I IFN has revealed some benefit, yet concerns over decline in quality of life and the toxicity of the treatment continue. As evidenced by the large number of clinical trials involving type I interferon for the treatment of cancer (more than 450 listed on https://clinicaltrials.gov, including some 50 trials that are recruiting patients at this time), study of this approach continues in order to better develop delivery methods, treatment regimens and identify those patients who are most likely to benefit.

### Inducers of endogenous type I IFNs

**Figure 3:**
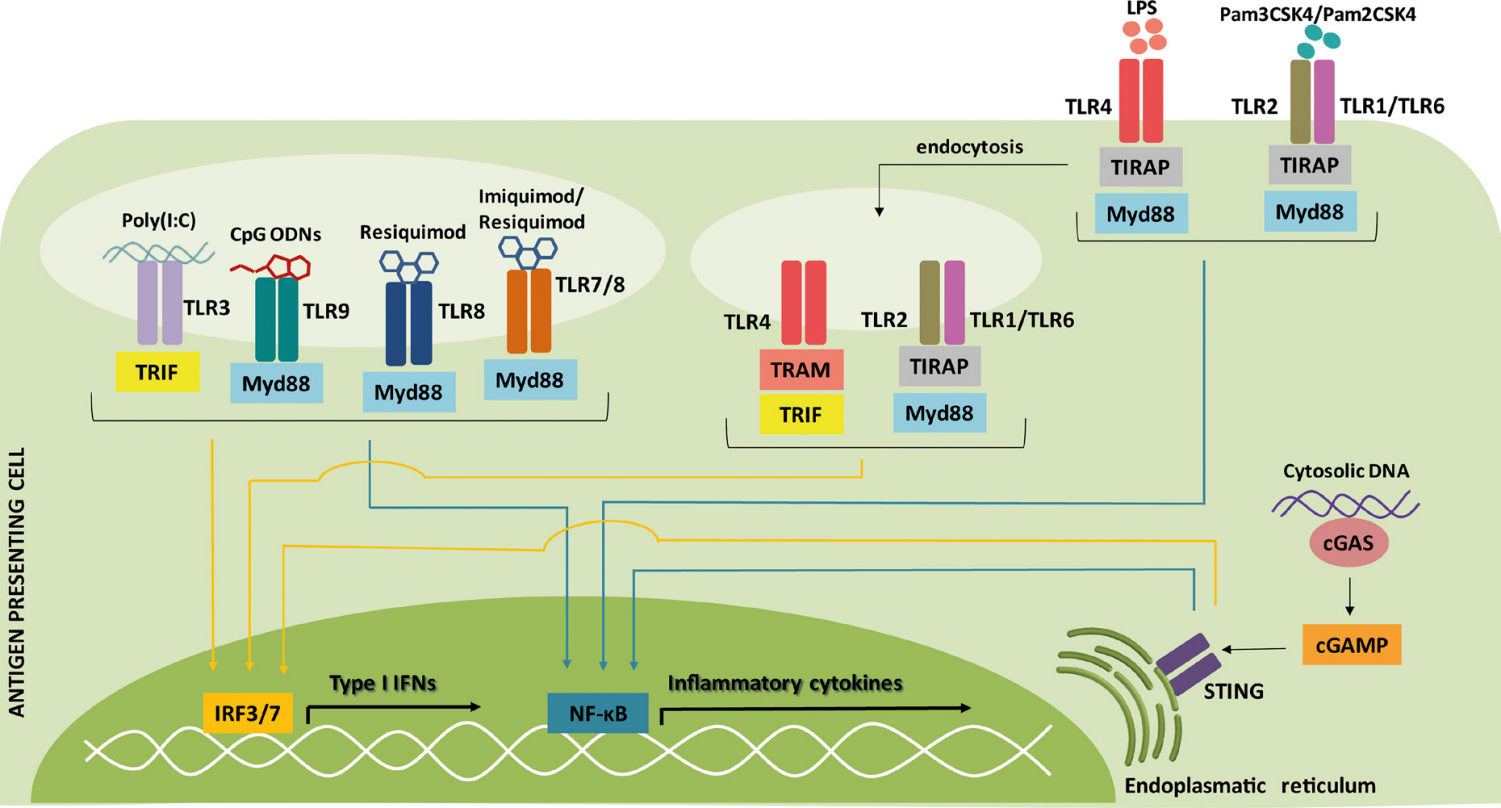
Signaling pathways of type I IFN inducers commonly used as adjuvants for cancer therapy Nearly all cells are capable of producing type I IFNs after sensing pathogen-associated microbial patterns (PAMPs) and danger-associated molecular patterns (DAMPs). This strategy is used to improve therapeutic cancer vaccines, increasing the immunologic response. The activation of pattern-recognition receptors (PRRs) leads to signaling through the adaptor molecules toll/interleukin-1 receptor domain-containing adaptor protein inducing interferon-β (TRIF) and/or Myd88, which culminates in the activation of IFN-regulatory factor 3/7 (IRF3/7) (yellow arrows) or nuclear factor-ĸB (NF-ĸB) (blue arrows) transcription factors and, consequently, in the expression of type I IFNs or inflammatory cytokines. Regarding the dendritic cell (DC) subtypes, the PRRs toll-like receptor (TLR)1, TLR2, TLR3, TLR4, TLR6 and TLR8 are expressed by monocyte-derived DCs and myeloid DCs, while TLR7 and TLR9 are only expressed by plasmacytoid DC. Lipopolysaccharide (LPS), triacylated lipopeptides (Pam3CSK4), diacylated lipopeptides (Pam2CSK4), polyribosinic-polyribocytidylic acid [Poly(I:C)], oligodeoxynucleotide (CpG ODN), TIR domain-containing adaptor protein (TIRAP), TRIF-related adaptor molecule (TRAM), 2′-3′-cyclic GMP-AMP (cGAMP), cGAMP synthase (cGAS), stimulator of interferon genes (STING).

The main purpose of cancer immunotherapy is to induce immune cells to effectively eliminate tumors, overcoming the immunosuppressive tumor microenvironment [204] and, as discussed here, the induction of type I IFNs may be a critical step towards this end. Indeed, in contrast with traditional vaccine adjuvants, such as aluminum compounds, that mostly stimulate humoral immune responses, targeting of DAMPs and/or PAMPs receptors to induce IFN-α/β is a very effective strategy for cell-mediated immunity and therefore an alternative as adjuvant in cancer vaccines (Figure 3). Here we explore the induction of endogenous type I IFN both as an adjuvant and as an immunotherapy on its own.

### Poly(I:C)

Polyribosinic-polyribocytidylic acid [Poly(I:C)] is a synthetic analog of double-stranded RNA, a ligand of the TRIF-dependent toll-like receptor-3 (TLR3) [205]. TLR3 is highly expressed in several tumors [206] and in immature myeloid DC, NK cells, T cells and macrophages [207, 208]. Poly-ICLC is a derivative of Poly(I:C) stabilized with poly-lysine (Hiltonol^®^, Oncovir Inc.) and indeed was shown to be 5- to 10-fold more resistant to hydrolysis [209]. Both are included in the National Cancer Institute’s ranking of immunotherapeutic agents with the highest potential of improving the cancer immunotherapy response [205].

After stimulation, TLR3 recruits adaptor protein TRIF and signals through activation of IRF3, NF-κB and activator protein-1 (AP-1), stimulating activation of the antiviral and pro-inflammatory responses [210].

*In vitro* studies have shown that Poly(I:C) induces maturation and activation of DCs, including enhancing cross-presentation [211], stimulates T cells [212] and NK cells [213], and induces secretion of pro-inflammatory cytokines by tumor and immune cells [214]. Poly(I:C) can also directly affect tumor growth and induce apoptosis of tumor cells [215], resulting in the availability of tumor-associated antigens (TAAs) for uptake by APCs.

In healthy volunteers, Poly-ICLC was shown to upregulate genes involved in the innate immune response including IFN-α, IFN-β, IFN-γ, the complement system and the inflammasome [216]. Poly-ICLC stimulated Th1 cytokines, increasing the Th1/Th2 ratio [217] and driving T cells toward a Th1 response. Sabbatini and colleagues have shown that 91% of patients with ovarian cancer in a poly-ICLC-vaccine cohort showed functional CD8^+^ T cell responses, compared to 25–62% of patients in non-poly-ICLC groups. Similarly, CD4^+^ T cell responses were stronger in patients treated with poly-ICLC-containing vaccines [218].

Interestingly, lymphocytes extracted from patients with persistent HPV infection were exposed to HPV16 virus-like particles (VLP) and then treated with Poly-ICLC, resulting in increased MHC class I and II, CD40, CD80, and CD86 expression and inducing HPV16 E7-specific CD8^+^ T cell responses *in vitro* [219].

Another version of Poly(I:C), Poly(I:C12U) (Ampligen®, Hemispherx Biopharma), has been used for chronic fatigue syndrome treatment [220] and activated moDCs, increasing the expression of surface MHC class I and II, CD83, CCR7, CD86, CD40 and IL-12 [221].

### Lipopolysaccharide

Lipopolysaccharides (LPS) from Gram-negative bacteria are TLR4 agonists. After LPS binding, TLR4 dimerizes and this is sensed by an adaptor molecule called toll/interleukin-1 receptor domain-containing adapter protein (TIRAP) [222]. Then, TIRAP recruits the signaling adaptor MyD88 and several interleukin-1 receptor-associated kinase (IRAK) family members [223], which activates inflammatory transcription factors such as AP-1 and NF-κB [224]. At the same time that TLR4 signaling is induced, several events take place to promote the TLR4 endocytosis. Upon delivery to endosomes, TLR4 recruits TRIF-related adaptor molecule (TRAM) and TRIF, which signals through a cascade of activated proteins in the cytosol and culminates in the induction of and IRF3 [225]. In this way, TLR4 engagement promotes the expression of pro-inflammatory cytokines and type I IFNs.

Several reports show that DCs generated from mobilized monocytes pulsed *in vitro* with TAAs and stimulated with LPS, with or without IFN-γ, were able to express IL-12 and CXCL10, polarize a Th1 immune response and may be useful for DC-based immunotherapy [226–230]. Interestingly, other studies have demonstrated that DCs generated using LPS are capable of inhibiting suppression mediated by CD4^+^CD25^+^Foxp3^+^ regulatory T cells [231] and that they restored CD4^+^ and CD8^+^ T cell proliferation, while DCs matured with a conventional cocktail (IL-1, IL-6, TNF-α, prostaglandin E2 - PGE2) did not fully restore T cell proliferation [232]. Also, the 3-O-deacylated monophosphoryl lipid A (MPLA) is a less toxic LPS derived from *Salmonella minnesota* R595 and used with alum in a prophylactic vaccine against human papillomavirus 16 and 18 (Cervarix^®^, GSK Vaccines) [233].

### Imidazoquinoline-like molecules

Imiquimod and Resiquimod are imidazoquinoline-like molecules that have been identified as TLR7/8 agonists based on their ability to induce DC maturation. TLR7 is mainly expressed in pDCs and, to some extent, in B cells and monocytes/macrophages [234], while TLR8 is primarily expressed in monocytes/macrophages and myeloid DCs [235]. Therefore, Imiquimod is used specifically to activate pDCs, inducing expression of IFN-α, IL-6, IL-8, IL-12 and TNF-α and stimulating a Th1 immune response; it has been approved by the FDA for treating basal cell skin cancer (Aldara^®^, 3M Pharmaceuticals) [236].

TLR7 also recognizes single-stranded RNA (ssRNA) derived from RNA viruses (like vesicular stomatitis virus, influenza A virus and human immunodeficiency virus) [237], synthetic poly(U) RNA and certain small interfering RNAs [238], thus pDCs are able to produce large amounts of type I IFN and cytokines in response to virus infection [237]. TLR8 recognizes Resiquimod and viral ssRNA and is upregulated after bacterial infection, having its highest expression in monocytes, although is expressed in several tissues [239].

These sensors utilize the universal adapter protein MyD88, which in turn activates the expression of IRF7 and NF-κB, thereby stimulating transcription of type I and III IFNs, inflammatory cytokines and chemokines [240], especially IFN-α, TNF-α and IL-12 [241]. Upon activation, pDCs also expresses the co-stimulatory molecules CD40, CD80 and CD86 and gain the ability to cross-present antigens in the context of MHC [242].

The use of Imiquimod brought to light intriguing observations regarding DC functions that are not usually considered. Drobits and co-workers showed that Imiquimod treatment promoted the secretion of both TRAIL and granzyme B resulting in pDC-mediated tumor killing [243]. Also, pDCs stimulated with agonists for TLR7 and 9 upregulated the surface expression of TRAIL in a type I IFN-dependent manner, causing the lysis of Jurkat cells and melanoma cell lines SKMel2 and WM793 [244].

The topical treatment of basal cell carcinoma, perianal Bowen's disease and superficial malignant melanomas with Imiquimod led to an increase in activated-pDC infiltration and to a reduction in neoplastic cells with complete regression in some cases [245–248].

Also, Imiquimod may have direct antitumor effects inducing apoptosis via modulation of the expression of Bcl-2/Bax [249–251] and autophagy [252, 253] in several cancer cells, as well as antiangiogenic properties, based on its induction of interferons, IL-10, and IL-12 [254], which end up inhibiting angiogenesis independently of their immunomodulatory functions. In fact, Imiquimod has been successfully used as an antiangiogenic agent to treat vascular proliferative lesions, such as infantile haemangioma, pyogenic granuloma and Kaposi’s sarcoma [254–256]. And, in a patient with melanoma, treatment with Imiquimod induced gene expression of angiogenesis or MMP inhibitors, like *IFN-*α, *KiSS1*, TIMP metallopeptidase inhibitor 1 (*TIMP1*), and thrombospondin 1 (*THBS1*), while decreasing expression of *bFGF* and *MMP9*, as shown by quantitative PCR of cutaneous melanoma metastasis biopsies performed before and after treatment [257].

### CpG ODNs

The unmethylated CpG ODN (oligodeoxynucleotide) TLR9 agonists are powerful adjuvants for the activation of pDCs. In humans, TLR9 is expressed only in pDCs and B cells [258] and recognizes unmethylated CpG motifs that are found in bacterial and viral genomes [239]. CpG ODNs are divided into four classes depending on the differences in their structure and immunoreactivity. Of these classes, almost all the CpG ODNs used in clinical trials have been class-B CpG ODNs (also known as K-type ODNs), however type I IFN is weakly induced by CpG-B ODNs. Class-A CpG ODNs (also known as D-type ODNs) have also been used but in fewer clinical trials. Class A and C CpG ODNs enter the lysosome compartments of pDCs and B cells to stimulate IFN-α production, while class B CpG ODNs enter the endosomal compartments of pDCs to induce their maturation [259].

CpG ODNs activate TLR9–MyD88–IRF7 and TLR9–MyD88–NF-κB signaling pathways of pDC to induce expression of MHC and costimulatory molecules such as CD40, CD80, and CD86, which results in CD4^+^ and CD8^+^ T cell maturation [260] and secretion of type I IFN [261] and IL-6, IL-12 and TNF-α [262]. Additionally, type I IFN and TNF-α secreted from pDCs activate NK and NKT cells [263].

A prospective Phase I trial with stage II–IV metastatic melanoma patients vaccinated with melanoma-associated antigen recognized by T cells-1 (MART-1) peptide, Montadine^®^ ISA-51 (an agonist made of mineral oil and surfactant from mannide monnooleate family [264]) and CpG 7909 showed that in the presence of CpG ODN there was 10-fold more MART-1 specific T cells induced in patients [265].

MelQbG10, which is G10 CpG ODN and the tumor peptides MART-1 coated with bacteriophage protein, was used in combination with Montanide^®^ ISA-51 and topical 5% Imiquimod cream in stage III/IV malignant melanoma patients. Patients vaccinated with MelQbG10 plus Montanide^®^ ISA-51 had significantly higher T cell induction versus MelQbG10 alone, but there were no significant differences in clinical outcome among the different treatment groups [266].

### Bacterial triacylated or diacylated lipopeptides

The fatty acid groups of triacylated lipopeptides are recognized by TLR2/TLR1 heterodimers [267], while the fatty acid groups of diacylated lipopeptides are ligands for TLR2/TLR6 heterodimers [268]. Previously, it was thought that TLR2/TLR1 and TLR2/TLR6 engagement elicited the pro-inflammatory pathway, but not the type I IFN responses [269]. It was shown that administration of TLR2 agonists can enhance effector and memory T cell responses, culminating in improved tumor rejection [270, 271]. TLR2 agonists can also increase expression of costimulatory molecules in B cell lymphoma, enhancing its sensitivity to NK and CD8^+^ T cells [272], or inducing caspase 8-dependent apoptosis [273].

However, recent studies have shown that bacterial ligands can induce type I IFN responses through TLR2 binding. After stimulation, the TLR2 heterodimers are internalized into endolysosomal vesicles, from which they induce IFN-β via MyD88 and IRF1/IRF7 [274] and this pathway requires TRAM [275], but is yet to be fully elucidated. In this way, TRAM acts as adaptor molecule for both TLR4 and TLR2, inducing IRF1 and IRF7 signaling from the endosome.

Dietrich and colleagues observed that stimulation of bone marrow derived macrophages (BMDMs) with Pam3CSK4 (synthetic triacylated lipopeptides TLR2/TLR1 agonist) and Pam2CSK4 (synthetic diacylated lipopeptides TLR2/TLR6 ligand) induced not just pro-inflammatory cytokines like TNF-α and IL-12, but also type I IFN-inducible genes, such as *CXCL-10*, *Mx2, IL-6* and *iNOS* [274].

SUP3 is a TLR2 agonist based on the structure of Pam3CSK4, but with a chemically more stable structure. SUP3 was shown to enhance cross-presentation by CD8^+^ cDCs *in vitro*, up-regulate the expression of CD40 and CD86 co-stimulatory molecules and induce production of IL-6 and TNFα in DC, culminating in an antigen-specific CD8^+^ T cell response and increased immunization against tumor challenge. SUP3 also induced antigen-specific antibodies such as IgM, total IgG and high affinity IgG [276].

### **2**′**-3**′**-Cyclic GMP-AMP**

Several authors have shown that 2′-3′-cyclic GMP-AMP (cGAMP) can be used directly as an adjuvant for antitumor therapy [277]. cGAS is a major sensor of cytosolic DNA, irrespective of the DNA sequence [278, 279]. Cytosolic DNA can trigger strong production of type I IFNs and other inflammatory cytokines in immune and non-immune cells. After DNA binding, cGAS undergoes a conformational change that promotes the conversion of GTP and ATP into cGAMP [280]. cGAMP then acts as a second messenger that activates the adaptor protein STING, at the endoplasmic reticulum membrane [279]. STING in turn activates the proteins inhibitor of nuclear factor-κB kinase (IKK) and TANK binding kinase 1 (TBK1), which activate NF-κB and IRF3, respectively, inducing production of cytokines and type I IFN [281].

DCs can also activate the cGAS–STING pathway after DCs phagocytose tumor cells and some of the tumor DNA escapes to the cytoplasm. Woo and co-workers (2014) showed that mice deficient for Myd88, TRIF, the purinergic receptor P2X7 (P2XR7), mitochondrial antiviral-signaling protein (MAVS) or retinoic acid inducible gene I (RIG) had no defect in priming of CD8^+^ T cells. Strikingly, in both STING-deficient and IRF3-deficient mice, there was a substantially diminished CD8^+^ T cell response against tumor-associated antigens and, in wild-type mice, transfer of tumor DNA to host APCs resulted in TBK1 and IRF3 phosphorylation and, as a consequence, production of IFN-β [282]. ECs are also producers of type I IFN in response to STING activation. Demaria and collaborators showed that intratumoral injection of exogenous cGAMP enhanced STING activation in the tumor microenvironment, resulting in stimulation of type I IFN response and antitumor CD8^+^ T cells, leading to growth inhibition of injected and contralateral tumors. Interestingly, this effect resulted mainly from tumor ECs, which were the main producers of IFN-β in response to cGAMP injection in both mouse and human [283].

In a study performed by Wang and co-authors, PD-L1 antibody was administered in a mouse model of melanoma and they observed that cGAS-deficient mice are refractory to the antitumor effects of a PD-L1 antibody. They showed a large increase of tumor-infiltrating leukocytes in wild-type mice after PD-L1 antibody treatment, but not in cGAS- or STING-deficient mice. This may be due to tumor cell killing caused by PD-L1 antibody treatment, which exposes tumor-associated antigens and DNA that are taken up by DCs. Then, tumor DNA escapes to the cytoplasm of DCs and activates the cGAS–STING pathway, inducing the production of type I IFN and the co-stimulatory molecule CD86, and activating a Th1 response. When they applied cGAMP intramuscularly, this caused inhibition of tumor growth and prolonged mouse survival after PD-L1 antibody treatment [284].

Previous studies have shown that intratumor injection of cGAMP and its analogs also induced antitumor effects. However, some authors suggest that STING activation may induce a suppressive tumor microenvironment and contribute to tumor growth and metastasis [285]. Metastatic brain cancer cells generate cGAMP, which is transferred by gap junctions to astrocytes, activating the STING pathway in these cells and producing proinflamatory cytokines, which in turn activate STAT1 and NF-**κ**B pathways in the metastatic cells, thus supporting tumor growth [286].

### Immunogenic cell death

During the last decade, the newly defined concept of immunogenic cell death (ICD) induced a thorough revision to the previously accepted, classic point of view cell death as a dichotomized phenomenon as either apoptotic, associated with a tolerogenic immune response that maintains tissue homeostasis or, in contrast, necrotic, a promoter of the inflammatory response [287]. Undeniably, along with the success of checkpoint blockade immunotherapy [288], ICD helped to cement the importance of the immune system during cancer treatment, especially pertinent when selecting which chemotherapy to administer since unsuccessful approaches are often immunosuppressive and unable to activate antitumor immunity.

ICD was originally demonstrated as a cellular and molecular response of cancer cells to anthracyclines that involves the exposure and secretion of immunogenic DAMPS, in a defined temporal sequence, providing both antigenic and stimulatory signals for the DC compartment to generate an effective CD8^+^ T cell attack against remaining tumor cells [287]. The first study that unveiled this mechanism showed that *ex vivo* treatment of MCA205 sarcoma cells with doxorubicin and subsequent inoculation of these dying tumor cells into naïve syngeneic mice protected them against a subsequent tumor challenge. Remarkably, this protection was not seen when cells were treated with mitomycin C (another chemotherapeutic agent) or when caspase-3 activity was blocked, showing that a specific property of cell death induced by doxorubicin was mediating immune stimulation. Furthermore, intratumoral application of doxorubicin in subcutaneous established tumors only exhibited therapeutic efficacy when treatment was performed in immunocompetent mice, whereas treatment in the nude background (lacking mature T cells) or in animals depleted of DCs abolished the immune response against the tumor cells [289].

Key mechanistic insights came later when Obeid and colleagues used a large panel of apoptosis inducers and identified changes in the plasma membrane proteome that were exclusively present in anthracycline treated cells and not in the presence of the pan-caspase inhibitor. Comparison of two-dimensional electrophoresis, followed by mass spectroscopic analyses, led to the identification of the endoplasmic reticulum chaperone calreticulin (CRT). Accordingly, knockdown of CRT negatively affected phagocytic uptake of dying tumor cells by DCs and abrogated the immune protection effect. Therefore, CRT release (ecto-CRT) was the first key feature identified as a determinant of the interaction of DCs and dying immunogenic cells and consequently the anticancer immune response [290].

Furthermore, as revealed in subsequent studies, other ICD determinants have been identified. This way, the proposed mechanism postulates that: (i) in response to lethal insult from doxorubicin treatment, dying tumor cells activate autophagy machinery and secrete ATP that, in turn, is recognized by purinergic receptors (P2RY2 and P2RX7) of DCs, promoting DC recruitment and activation; (ii) exposition of Annexin A1 mediates DC contact with the dying tumor cell; (iii) secretion of CRT, as a consequence of endoplasmic reticulum stress, acts on antigen uptake by DCs; (iv) release of the alarmin high-mobility group box 1 (HMGB1) from the nucleolus, which then binds to TLR4 of DCs to induce full maturation status, secretion of IL-1β and eventually leading to priming of T cells with complete cytotoxic capacity [291].

Interestingly, all of these processes regarding release and secretion of ICD markers alone or in combination cannot predict with certainty if the cell death process will truly be immunogenic, which suggests that additional, unknown factors remain to be identified [292]. Along these same lines, recently a novel mechanism was uncovered: the cancer cell–autonomous secretion of type I IFNs [293].

The role of type I IFNs in ICD was revealed by analyzing immunologically relevant transcriptional changes induced in sarcomas upon intratumoral treatment with doxorubicin. Among the modulated pathways, transcripts associated with response to viral infections were indicated by type I IFN stimulated-genes, pointing to a type I IFN fingerprint in the cancer cells. Indeed, antibody mediated blockade of IFNAR1 or IFN-α/β neutralization markedly inhibited doxorubicin’s antitumor effects. Sarcoma tumors derived from an *Ifnar2*^*−/−*^ background did not respond to doxorubicin when transplanted into a wild-type host, suggesting that the IFN-β produced during ICD was most likely affecting the tumor cells, not the host immune system. As further demonstrated, *Ifnar1*^*−/−*^ tumor cells failed to secrete CXCL10 in response to doxorubicin, just as was also seen in tumor cells derived from *Tlr3* KO or its adaptor *Trif*. These data demonstrated that ICD inducers act by stimulating IFN-β secretion through an autocrine and paracrine mechanism that takes place upon TLR3 recognition of self RNA from dying cells, activating the CXCL10-CXCR3 signaling axis to attract effector immune cells [293].

In accordance with these findings, radiation therapy (RT), a known ICD inducer, was also reported to depend on IFN-β signaling. In the work of Burnette and collaborators, local ablative RT of B16-SIY tumors resulted in striking tumor regression and local production of IFN-β by tumor-infiltrating CD45^+^ cells. In agreement, tumor associated DCs presented higher levels of maturation markers (CD40, CD80 and MHC-I and II molecules) after RT, yet if treatment was performed in an *Ifnar1* KO background, therapeutic control was completely lost. Additionally, to determinate the host compartment in which IFN-β was necessary, bone marrow transplants from *Ifnar1* KO mice to a WT host revealed a requirement for the hematopoietic cells, more specifically the CD11c^+^ and CD11c^-^ myeloid populations, that in a context lacking IFN-β lost their cross-priming capacity within the tumor microenvironment [294].

Another ICD inducer that relies of type I IFN activity is the oncolytic Newcastle Disease Virus (NDV) [295], which is an anticancer virotherapy strategy, and as such, was expected to be impaired by the antiviral properties of IFNs, thus negatively affecting therapy outcome. However, as demonstrated in the work of Zamarin and colleagues, intratumoral applications of the oncolytic NDV accompanied with CTLA-4 checkpoint blockade promoted complete regression of B16 tumors in both primary and non-treated secondary sites, showing a remarkable systemic immune protection. Additionally, the authors found that this immune protection was mediated by both NK and CD8^+^ T cells, and, unexpectedly, if treatment was performed in a *Ifnar1*^−/−^ host, therapeutic efficacy was abrogated in the injected tumors as well as in the contralateral challenge, even when combined NDV plus CTLA-4 treatment was applied [296].

Three pathways of innate immune sensing can lead to *Ifnb* gene transcription: (i) TLR stimulation signals through MyD88 and TRIF adaptors, (ii) RIG-I senses cytosolic RNA and signals through the adaptor protein IPS-1, and (iii) STING senses cytosolic DNA and promotes type I IFN expression [297, 298]. Consequently, induction of type I IFN in the tumor microenvironment correlates with T cell infiltration. Gajewski demonstrated, through melanoma gene expression profiling, that tumors infiltrated with CD8^+^ T cells also exhibited a type I IFN transcriptional signature [299], a suggestion that type I IFN signaling might participate in innate recognition of tumors [157]. Also, as said before, mice deficient in type I IFN response showed decreased spontaneous T cell priming in transplantable tumor models and increased tumor induction using methylcholanthrene [6].

Overall, the findings presented here highlight the critical role of integrated innate and adaptive immune responses in order to achieve full therapeutic efficacy and, most importantly, revealing type I IFN signaling as an indispensable propeller of the cancer immunity cycle. Yet, it remains to be determined which tumors are likely to benefit from ICD treatment, for example, one report indicates that spontaneous mammary tumors in (MMTV)-NeuT transgenic female mice can successfully respond to immunogenic chemotherapy even in the absence of the immune system [300]. But, as shown by Pfirschke and collaborators, pretreatment with the combination of oxaliplatin-cyclophosphamide can increase T cell infiltration in resistant *Kras/Trp53* mutant tumors, rendering them sensitive to anti-PD-1 and CTLA-4 blockade [301], suggesting the potential benefit of associating chemo and immunotherapy. However, inducing ICD using multiple inflammatory immunotherapeutic agents may not boost immune attack, since negative regulatory mechanisms will likely be stimulated and, thus, impede the immune response.

## TYPE I IFNS IN CANCER GENE THERAPY: TARGETING TUMOR AND DENDRITIC CELLS

According to the Journal of Gene Medicine, 2409 clinical trials making use of gene transfer approaches have been included in their online database since 1989, of which 1554 (approximately 64.5% of the total) were aimed at treating cancer. Cancer gene therapy is expected to remediate faulty gene function in order to kill tumor cells or render them susceptible to killing by chemo/radiotherapy or immune attack. Such approaches may include the silencing of oncogenes [302], the transfer of tumor suppressor genes, generally aiming to trigger mechanisms of cell death [303], and the transfer of immune modulating genes in order to elicit antitumor systemic responses [304, 305]. As will be discussed further, the gene therapy approach may not target the tumor cell directly, but can be used to trigger an anti-tumor immune response, such as in the case of vaccines based on modified DCs.

Many vectors have been employed for the transfer of type I IFN genes, including for modification of tumor cells or DCs (Table 1). Among these vectors, adenovirus, AAV and, more recently, non-viral liposome mediated gene transfer have been used in both basic and clinical research protocols [306–311]. Specifically in the case of IFN-α gene transfer, adenovirus is one of the most commonly employed vectors for *in situ* treatment models, yet lentivirus is better suited for *ex vivo* creation of IFN-α secreting cells.

**Table 1:** Properties of the main vectors used in gene therapy protocols

**Vector**	**Integrative**	***in vitro* delivery efficiency**	***in vivo* delivery Efficiency**	**Capacity to trigger immune response**	**Efficient production**
Adenoviral	No	High	High	High	Yes
Lentiviral	Yes	High	High	Low	No
Retroviral	Yes	High	High	Low	No
AAV	No	High	High	High	No
Liposomes	No	High	Low	Low	Yes

Abbreviations: AAV, Adeno-associated virus.

In preclinical models, as expected from its antitumor functions, IFN-α gene transfer induced: (i) cell cycle arrest, (ii) apoptotic cell death mechanisms [312–314], (iii) decreased hemoglobin index and microvessel density, and (iv) necrotic ischemia in tumor tissue [312, 313, 315]. Regarding the immunomodulatory property of IFN-α, it was also observed that the gene transfer, alone or combined with other therapy approaches, lead to: (i) increased presence of infiltrating CD8^+^ and CD4^+^ T cells and decreased Foxp3^+^ cells in the tumor parenchyma and also augmented MHC-I expression on tumor cells [316]; (ii) enhancement of NK cell cytolytic activity [312] and (iii) increased presence of CD11c^+^ cells in regional lymph nodes [317]. In this way, the immunosuppressive features of the tumor microenvironment can be overcome upon IFN-α gene transfer and indications of a systemic antitumor immune response are uncovered.

More recently, the effect of the IFN-α on metastasis was investigated. In this approach, hematopoietic stem cells (HSCs) were modified with a lentiviral vector in order to generate Tie2^+^ macrophages/monocytes that constitutively express IFN-α. The natural homing of these cells to tumor sites was observed, leading to a reduction in hepatic metastases from colorectal cancer, without exerting a negative influence on the homeostasis of hematopoiesis [318, 319].

In light of the successful results obtained from IFN-α gene transfer to cancer in basic-research models, a phase-I clinical trial was carried out using a recombinant adenoviral vector to treat non-muscle invasive bladder cancer in which 17 patients were enrolled. The subjects received escalating doses of the vector and the gene transfer efficacy was assessed by examining cytokine levels in the urine. Even at the highest dose, the treatment was well tolerated, with only mild adverse events being observed. Regarding the treatment efficacy, it was reported that 7 patients achieved complete response at 3 months [320].

In a similar way, in basic research protocols aiming to assess the effects of IFN-β gene transfer in both *in vitro* and *in vivo* models, it was observed that the treatment: (i) reduced tumor cell proliferation [133, 321–323]; (ii) decreased cell viability in both monolayer and spheroids cultures [308, 322]; (iii) increased long-term survival with reduced tumor burden [311, 323–327] and (iv) reduced tumor volume without notable toxicity, yet an increase in apoptotic cells and areas of necrosis in tumor tissue [133, 321, 324]. In this way, IFN-β gene transfer may offer an advantage, localized high concentrations of this protein, which cannot be achieved with biochemotherapy.

Delving into the effect of gene transfer on the microenvironment and immune system, in preclinical models after treatment it was reported that: (i) the cells presented downregulation of genes associated with angiogenesis, such as *bFGF*, *MMP9*, *VEGF-A* and *IL-8* [311, 322]; (ii) there was decreased quantity and density of blood vessels and diminished levels of hemoglobin in the tumor [311, 327]; (iii) treated animals were less prone to develop spontaneous metastasis, became resistant to a second tumor challenge or to the establishment of induced metastases [323, 326, 327]; (iv) increased infiltrating CD8^+^ T lymphocytes [323, 326, 328], activated NK cells [326, 329] and macrophages [133, 324, 329] as well as increased levels of MHC-I on the tumor cells [326]. The efficacy of IFN-β gene therapy was considerably decreased only in animals depleted of CD8^+^ T cells, indicating that this class of lymphocytes play a critical role in the immunomodulation stimulated by IFN-β [328].

Although many vectors are being used for gene transfer, armed oncolytics carrying IFN-β has recently gained ground. Approaches using the Vesicular Stomatitis Virus (VSV) encoding IFN-β have been shown to elicit a strong antitumor immune response, decreasing infiltrating T-reg cells and increasing CD8^+^ cells, and also stimulating the expression of PD-L1 on tumor cells [330]. Since VSV-IFN-β offers increased capacity to elicit both innate and adaptive immune responses as well as preferential replication in tumor cells, it is safer and more effective as compared to VSV with no transgene [331, 332], features that led to the establishment of a phase I clinical trial in 2012, that is still ongoing with estimated primary completion date in June 2017 (https://clinicaltrials.gov/ct2/show/study/NCT01628640).

Despite the positive responses seen in preclinical studies, only a few clinical protocols using recombinant vectors for the delivery of IFN-β in cancer patients have been carried out [333–335]. As an example, in a phase I clinical trial using a recombinant adenoviral vector, 11 patients with recurrent malignant glioma received different doses of the vector by stereotactic injection in the tumor site. The first injection was performed approximately one week before the scheduled tumor resection surgery and a second right after the procedure. After the treatment regimen it was clear that the vector treatment reproduced some data from the preclinical models, i.e, induction of apoptosis and the presence of necrotic areas in the treated tissue. Although a treatment-related dose limiting toxicity was seen in one patient enrolled in the highest dose cohort, IFN-β gene therapy was shown to be a safe and potentially effective approach [336]. On the other hand, despite its proven safety, it was also seen in another clinical trial that repeated doses of the adenoviral vector did not considerably improve the clinical outcome in patients with mesothelioma due to the fast development of neutralizing antibodies against the vector, an obstacle that, perhaps, could be circumvented by using a non-immunogenic vector or by combining gene transfer with additional therapeutic approaches [332].

### Modified dendritic cells expressing type I interferons: crossroad between cancer vaccines and gene therapy

In order to induce a host immune response against tumor cells, genetically modified DCs have been used in vaccination protocols. In spite of the clear rationale, DC-based vaccination faces some technical obstacles, such as the best condition for their activation such that the antitumor immune response is efficiently induced [337]. Given the influence of type I IFNs on DCs, it is reasonable that IFN-α/β gene transfer be used to activate DCs and positive results have been reported. For example, mice bearing GL261 glioma tumors were treated with one intratumoral injection of an adenoviral vector encoding IFN-α followed by implantation of syngeneic bone marrow-derived DCs resulting in increased survival due to an antitumor immune response dependent on CD8^+^ T cells, and it was shown that the animals acquired a certain level of resistance against a second tumor challenge [338]. In another model the vaccination protocol consisted of intratumoral delivery of DCs previously modified with a recombinant adenoviral vector encoding IFN-α in combination with irradiated tumor cells engineered to express IL-4 or GM-CSF, resulting in stronger tumor specific CTL responses in the cervical lymph node and increased survival [339].

Specifically regarding the genetic modification of DCs, many recombinant vectors have been used, such as those derived from adenovirus, lentivirus, retrovirus, AAV and Sendai virus [340]. In spite of the many types of vectors available for the genetic modification of DCs, adenoviral vectors are most commonly used since they provide highly efficient gene transfer and expression, easy handling and high-titer preparations. Even so, adenoviral vectors present some disadvantages, e.g. pre-existing neutralizing antibodies and transductional dependency on the coxsackievirus and adenovirus receptor (CAR) [341]. However, the former should not present a barrier when virus is applied to DCs *ex vivo* and the latter can be overcome with the use of modified adenoviral vectors that no longer depend on CAR. Still, adenoviral vectors trigger molecular mechanisms leading to the maturation of DCs and, as a consequence, a more consistent antitumor immune response [342, 343].

In addition to adenovirus, other vectors have been gaining space in the field of genetically modified DCs. For example, non-viral methods are being more frequently used, including mRNA transfection, due to their lower manufacturing costs, comparable levels of expression, and relative transfection efficacy, especially with the use of electroporation [344]. Another emerging viral vector is the Sendai virus due to its high transduction efficiency *ex vivo* and *in vivo*, augmented capacity to trigger antitumor immune responses and elicit DC maturation [345, 346]. Along the same lines, exploring the properties of IFN-α produced by pDCs, infection of pDCs with a replication-deficient herpes simplex virus 1 (HSV-1) *d*106S vaccine strain showed a robust cytotoxic effect against various melanoma cell lines that was equivalent or superior to the effects induced by synthetic TLR7 and TLR9 agonists [347].

### p19Arf and interferon-β combined gene transfer

As presented above, gene transfer of IFN-α/β directly into the tumor mass or into dendritic cells presents promising results that merit further development for clinical application. However, if one aims to increase the intrinsic antitumor and immunomodulatory properties of type I IFNs, which strategy should be used? More specifically, using a gene transfer method, how can we assure that most of the transduced tumors cells would die? Since type I IFNs are already considered sufficiently immunogenic, can we enhance their immune stimulation?

In light of these questions, our lab has previously developed a set of unique adenoviral vectors which utilize a p53 responsive promoter, called PGTxβ [348] to direct the expression of the cDNAs for p19^Arf^ (p19^Arf^ for mouse and p14^ARF^ for humans) and for IFN-β. Arf is a tumor suppressor protein that is encoded by the CDKN2A locus (also encoding the p16^INK4a^ protein) [349] and is mainly known for being a functional partner of p53, since after oncogenic stress Arf associates with MDM2 and prevents MDM2 mediated ubiquitination of p53 for posterior degradation [350]. Thus, Arf can enable p53 to trigger growth arrest, apoptosis and also acts in a p53 independent manner by inhibiting ribosomal RNA processing, promoting apoptosis and regulating autophagy [351, 352].

By combining Arf and IFN-β along with the p53 responsive promoter, we hoped to create interplay between: (i) transgene control, (ii) p53/Arf pro-apoptotic functions and (iii) IFN-β antiviral and immunomodulatory activities. Indeed, other studies have already pointed potential benefits of targeting the p53/Arf/IFN-β pathways, but never explored its therapeutic application. For example, Takaoka and collaborators have shown that IFN-α/β activates p53 transcription and stabilizes its protein levels [353]. Interestingly, they showed that p53 and type I IFNs work cooperatively to potentiate the apoptotic machinery and mediate tumor suppression and viral control functions. Furthermore, Sandoval and colleagues showed that apoptosis induced by type I IFNs requires p14Arf, but not p53, since human sarcoma cells null for p14^ARF^ undergo apoptosis when p14^ARF^ is reintroduced in the presence of IFN-α/β, but the same observation is not seen with p53 [354].

Based on this evidence, we decided to explore the murine B16F10 (B16) melanoma cell line as a model since it harbors p53 in its wild type form, as seen in 90% of human melanoma cases [355] and is a well-known model for immunotherapies. Remarkably, in our initial observations we noticed that combined gene transfer of p19^Arf^ and IFN-β, but not the either single treatment, provoked massive cell death while up-regulating p53 target genes *p21*^*Waf1*^, *Mdm2* and *Puma* [356].

Evidence for superior immune stimulation came from two distinct immunization contexts. In the first, mice were vaccinated prophylactically with *ex vivo* transduced B16 cells that while dying were inoculated in naïve syngeneic C57BL/6 mice and seven days later, mice were challenged with fresh B16 cells in the contralateral flank. Tumor formation was completely abrogated at the vaccine site in hosts with competent NK cell compartment due to the up regulation of *Il-15*, *Ulbp1* NK cell receptor, *Killer/Dr5* and *Fas/Apo-1* death receptors on the treated cell, thus providing a safety benefit for the combination. At the challenge site, a dramatic decrease in tumor progression was observed and was dependent on tumor-infiltrating CD4^+^ and CD8^+^ T lymphocytes. Unexpectedly, in this prophylactic model, IFN-β alone or in combination with p19^Arf^ showed similar protection and T cells presented similar killing capabilities and levels of IFN-γ and TNF-α secretion. This would argue that there was no evident immunological superiority for the combination. However, when exploring a therapeutic tumor model, where the tumor challenge was made before the immunization step, only p19^Arf^/IFN-β vaccinated mice displayed reduction in tumor progression [357].

In support of this evidence, now in the second immunization context, mice bearing heterotopic (s.c) lewis lung carcinoma (LLC1) tumors were treated with four rounds of adenoviral injections directly into the tumor mass and subsequently challenged with fresh LLC1 cells in the opposite flank. Remarkably, mice that had their primary tumor treated with the p19^Arf^/IFN-β combination showed improved tumor control at the challenge site even when compared to IFN-β single treatment, showing superior immune protection by the combined p19^Arf^ and IFN-β *in situ* gene therapy [358].

Furthermore, seeking to gain mechanistic insights on how the combination could induce cell death and immune stimulation, we evaluated the transcriptional profile of critical protein pathways, revealing that only the p19^Arf^/IFN-β combination induced genes related to both the p53 pathway and apoptosis as well as IFN-β immune response and antiviral functions [359]. We also noted that the use of the adenoviral vector was a critical component for inducing cell death, reinforcing the antiviral aspect of the response. Intriguingly, the p19^Arf^/IFN-β combination promoted cell death by a different mechanism than that seen for the individual treatments, since inhibition of capase-3/7 increased the levels of cell death upon the individual treatment with p19^Arf^ or IFN-β, but did not affect the p19^Arf^/IFN-β group, suggesting that a caspase-independent mechanism of cell death was induced by the combined treatment. Of the three groups, the combination showed the lowest caspase 3 activity, while displaying features of necroptotic death, as revealed by the increase of Rip-3 (key mediator of necroptosis) and TNF receptor (*Tnfrsf1A*, an activator of the necrosome complex). Moreover, consistent with the recent demonstration that necroptosis can promote ICD [360], only the combined gene transfer of p19^Arf^ and IFN-β was accompanied by the exposition of calreticulin, ATP secretion and HMGB1 release, providing mechanistic support for the immunomodulatory superiority of the combination [359].

Taken together, we believe that our data provide functional and mechanistic evidence to classify our p19^Arf^ and IFN-β combined gene transfer as a novel agent for cancer immunotherapy. In fact, to the best of knowledge, no other gene transfer strategy employing non-replicating viral vectors has been shown to unleash ICD. Although we have identified NK cells, CD4^+^ and CD8^+^ T lymphocytes as critical cell mediators, we do not fully understand the mechanism by which these cells cooperate to bring about the anititumor immune response, most importantly we have not yet analyzed how DCs are being affected. Since NK cells can assume a helper phenotype to modulate DC priming function [178], it will be interesting to investigate how the NK cells activated in the vaccine site are interacting with DCs and promoting antigen uptake in our approach. Along the same lines, we expect that the p19^Arf^/IFN-β combination will provide not only an IFN-β immunomodulatory stimulus, but also immunogenic DAMPs unleashed during the ICD process, which together may provide an ideal stimulus for DC maturation, especially in the immunosuppressive tumor microenvironment. Another possibility regarding DCs would be to generate *ex vivo* derived DCs and use treated dying tumor cells as adjuvant as well as a source of antigen, an application that was successfully demonstrated in a pre-clinical model of high-grade glioma tumors [361]. Nevertheless, despite the long road ahead, IFN-β gene transfer and its combination with Arf holds a promising position in the cancer immunotherapy field.

## CONCLUSIONS

**Figure 4:**
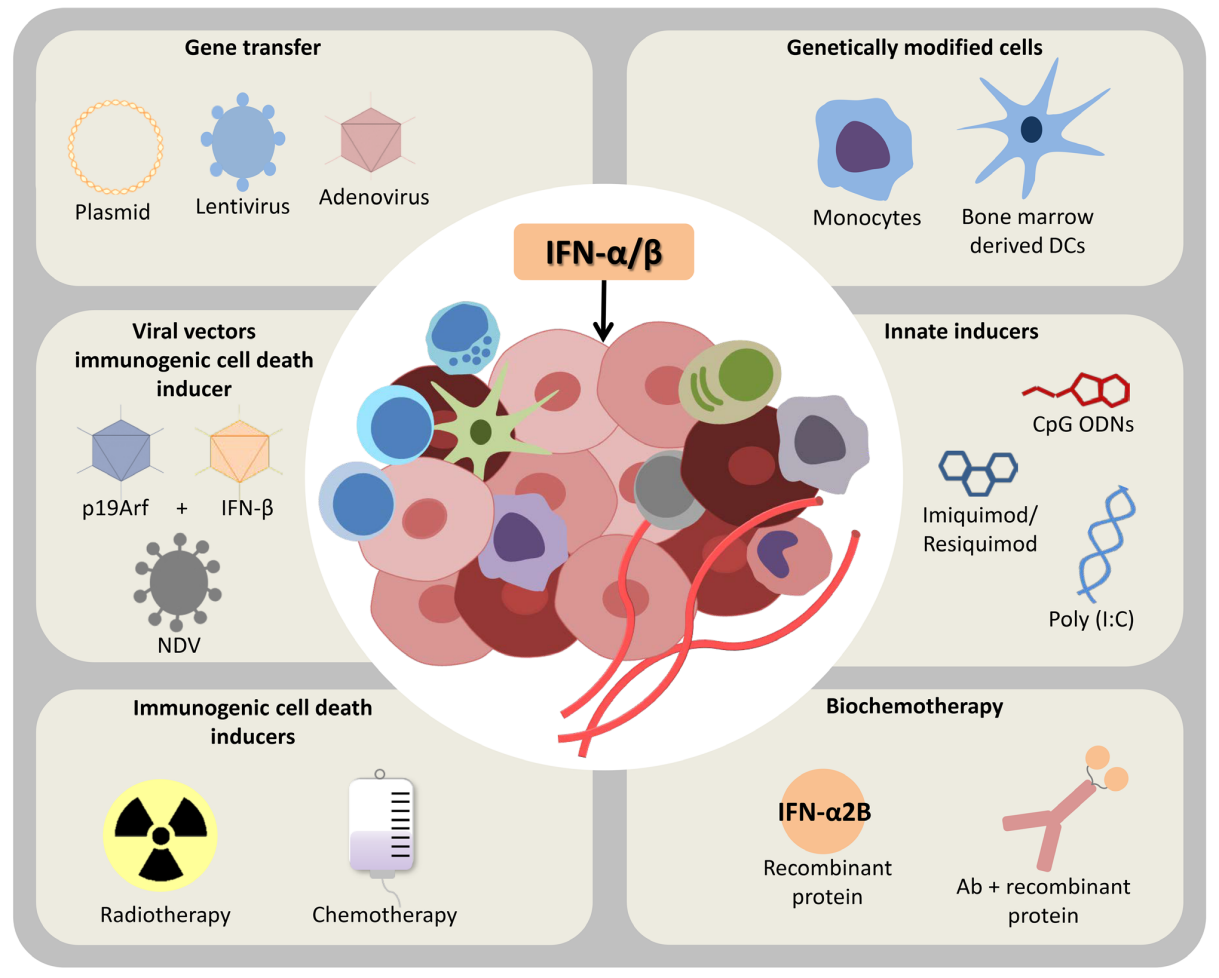
Harnessing type I interferons in cancer therapy During the last decades several strategies have been developed in order to exploit the antitumor properties of type I interferons (IFNs) in the tumor microenvironment. Indeed, diverse strategies range from the stimulation of tumor cells to produce their own IFN-α/β [e.g., inducers of immunogenic cell death, agonists of toll like receptors (TLRs) and gene therapy] or to deliver it to the cancer microenvironment, for example recombinant protein or dendritic cells (DCs) modified *ex vivo.* Though there is no consensus on which strategy is likely to provide the best results and much more remains to be understood concerning type I IFN’s pleiotropic functions, its combination with other treatment modalities, such as checkpoint blockade immunotherapy, is expected to unleash the full force of the immune system against cancer. Newcastle disease virus (NDV), antibody (Ab), oligodeoxynucleotide (CpG ODNs), polyribosinic-polyribocytidylic acid [Poly(I:C)].

Type I IFNs certainly play a critical role in the anti-cancer immune response and represent attractive strategy for therapy. Indeed, considering immunomodulation of the tumor microenvironment and its components, including stroma, immune and tumor cells, type I IFNs can be exploited by several strategies, such as inducers of ICD, agonists of TLRs, gene therapy and recombinant protein for the treatment of cancer (summarized in Figure 4). However, as discussed above, owing to their complex regulatory mechanisms, depending on the model of study, therapeutically induced type I IFNs have been shown to be required for either tumor cells or for infiltrating immune cells, especially DCs, to affect an anti-tumor response. Studies that can obtain deeper mechanistic insights are surely needed to clarify this dual requirement. For example, using a therapeutic model of systemic poly A:U application, Nocera and colleagues have visualized IFN-β in the tumor microenvironment, identifying the CD11c^+^ population as the main host source of IFN-β, but not the only one [362]. In this model, host type I IFN signaling was absolutely required for therapeutic efficacy and for poly A:U induced antitumor immunity. Moreover, using the same IFN-β luciferase reporter mouse, Lienenklaus and collaborators have previously revealed tissue-specific expression of IFN-β following infection with influenza or La Crosse virus, but most importantly, that IFN-β is constitutively expressed in low amounts by several tissues, including thymic epithelial cells, to maintain an activated state prepared for infection by pathogens [363].

Nevertheless, having in mind the central position of DCs in the cancer immunity cycle and the profound immunomodulation exerted by type I IFNs, even in the scenario that type IFNs are mainly impacting tumor cells, it would be reasonable to expect that antigen presenting functions of DCs are also being affected. Moreover, the dynamics and expression levels are also important factors to be considered, for example, should a tumor that already displays an ISG signature or a CD8^+^ T cell infiltrate be treated with the same amount of type I IFNs as compared to tumors that do not? Along the same lines, taking in to consideration the adaptive resistance mechanisms observed both in tumor and host cells, would an IFN based treatment in an inflamed tumor simply favor immunosuppression [184]? Some of the other major hurdles that must be overcome include the toxicity seen when high-dose recombinant protein is administered systemically as well as the relatively bland response encountered when IFN-α/β are applied as single agent gene therapies. We and others propose that more sophisticated ways to deliver a more localized concentration of type I IFN along with a tighter control over expression dynamics would alleviate adverse effects while still providing the desired biological effect. Gene therapy continues to be a promising method for the delivery of IFN-α/β in such manner, though we have strong evidence that the delivery of a second factor may be critical to releasing the full force of ICD. Even so, more experimentation is necessary to identify novel partners for IFN-α/β, including their pairing with chemo/radiotherapy and checkpoint blockade. Key regulators of the interplay between IFN-α/β, DCs and immune activation are still being revealed and, we propose, will continue to play an ever more critical role in cancer therapy.
